# Increased RNA editing in maternal immune activation model of neurodevelopmental disease

**DOI:** 10.1038/s41467-020-19048-6

**Published:** 2020-10-16

**Authors:** Hadas Tsivion-Visbord, Eli Kopel, Ariel Feiglin, Tamar Sofer, Ran Barzilay, Tali Ben-Zur, Orly Yaron, Daniel Offen, Erez Y. Levanon

**Affiliations:** 1 Sackler Faculty of Medicine, Human Molecular Genetics & Biochemistry, Felsenstein Medical Research Center, Tel-Aviv University, Tel Aviv, Israel; 2 The Mina and Everard Goodman Faculty of Life Sciences, Bar-Ilan University, Ramat Gan, Israel; 3 Department of Biomedical Informatics, Harvard Medical School, Boston, MA USA; 4 Departments of Medicine and of Biostatistics, Harvard University, Boston, MA USA; 5 Lifespan Brain Institute Children’s Hospital of Philadelphia and Penn Medicine; the Department of Child and Adolescent Psychiatry and Behavioral Sciences, CHOP, Philadelphia, PA USA

**Keywords:** RNA editing, Neurodevelopmental disorders

## Abstract

The etiology of major neurodevelopmental disorders such as schizophrenia and autism is unclear, with evidence supporting a combination of genetic factors and environmental insults, including viral infection during pregnancy. Here we utilized a mouse model of maternal immune activation (MIA) with the viral mimic PolyI:C infection during early gestation. We investigated the transcriptional changes in the brains of mouse fetuses following MIA during the prenatal period, and evaluated the behavioral and biochemical changes in the adult brain. The results reveal an increase in RNA editing levels and dysregulation in brain development-related gene pathways in the fetal brains of MIA mice. These MIA-induced brain editing changes are not observed in adulthood, although MIA-induced behavioral deficits are observed. Taken together, our findings suggest that MIA induces transient dysregulation of RNA editing at a critical time in brain development.

## Abstract (web summary)

Interferon can be induced by the viral mimic PolyI:C, which can in turn activate RNA editing enzymes. Here the authors identify transient changes in A-to-I RNA editing following maternal immune activation in mice.

## Introduction

The etiology of major neurodevelopmental disorders (MND) such as schizophrenia and autism still remains a mystery. Current evidence suggests that a combination of polygenic susceptibility and exposure to environmental risk during sensitive periods of brain development can impair neurodevelopment, and confer vulnerability to the development of MND^1–5^. Possible environmental risk factors for MND include intrauterine stressors, such as maternal bacterial and viral infections during pregnancy^6–10^.

Maternal immune activation (MIA) rodent models that are based on this observation involve exposing a pregnant rodent to specific viral pathogens, immune-stimulating agents, or pro-inflammatory cytokines, and evaluating the subsequent long-term brain and behavioral effects in the offspring^11^. Infection-induced disruption of early fetal brain development was shown to significantly affect postnatal brain development and maturation, and to lead to structural and functional deficits that are dependent on postnatal maturational processes^12,13^. MIA models therefore possess both the construct and face validity to study MND, and are pivotal in the quest to elucidate the underlying biology responsible^11,13,14^. However, despite much progress, the biological mechanisms through which MIA exerts a long-term deleterious effect on brain development and function remain unknown.

The viral mimic polyriboinosinic–polyribocytidilic acid (PolyI:C) has been shown to induce a multitude of behavioral, cognitive, and pharmacological abnormalities^13,15,16^. PolyI:C is a commercially available synthetic analog of double-stranded RNA, which is recognized by the mammalian immune system as foreign, due to its similarity to viral structures^17^. Systemic administration of PolyI:C leads to induction of the innate immune system, by activation of dsRNA sensors, such as melanoma differentiation-associated protein 5 (MDA5) and Toll-like receptor 3 (TLR3)^18^. These in turn, lead to the expression of innate immune response genes and proteins, as well as an induction of the interferons IFNα and IFNβ^16,19^.

Among its many biological effects, interferon (IFN) is responsible for the transcription of the Adenosine Deaminase Acting on RNA (ADAR) protein (also known as ADAR1) in its IFN inducible form, the p150 isoform^20^, present both in the nucleus and in the cytoplasm^21^. The ADAR family of enzymes carry out the common post-transcriptional modification, A-to-I RNA editing, in which ADAR deaminases adenosine to inosine^22–24^. This editing is applied to long double-stranded RNA transcripts, which are recognized by MDA5 in the cytoplasm^25^, or to RNA elements in the nucleus. Since inosine is recognized by the ribosome as guanosine during translation, editing can alter the proteome outcome.

The magnitude of RNA editing by ADAR proteins is immense, with millions of sites already identified in the human genome^26–28^, as well as in the genomes of many other organisms^29^. Alterations in editing levels are associated with a number of neurological disorders, including dementia and epilepsy^30,31^. The vast majority of A-to-I RNA editing activity occurs in non-coding primate-specific *Alu* repetitive elements^32–35^. Even though most of these sites occur in non-coding regions of the genome, they appear to be essential for development^36^. Notably, there is an enrichment of coding sites in neural tissues. These are predominantly edited by ADAR2 (refs. ^37,38^), and since many known editing sites are located within genes involved in brain function, their editing may also produce a phenotypic effect^37^. In spite of the fact that brain related lethality is more associated with deficiency of ADAR2 rather than ADAR1, both ADAR enzymes share editing activity, and may compete for certain substrates. As a corollary, each ADAR is able to compensate for a deficiency of the other and in a few cases can even boost the normal levels of editing at the other’s sites^39–41^. This tight interplay implies that alterations in ADAR1 activity may also be important for proper brain function and development.

Despite the possible involvement in brain development, probably the most important function of ADAR1-mediated editing of endogenous double-strand RNA (dsRNA) is to prevent an inappropriate cytosolic innate immune system response^42–44^. Editing of dsRNA, a structure commonly formed by viruses, prevents binding of dsRNA sensors such as MAD5 (refs. ^42–44^), thus averting an IFN response that could severely damage host cells. Furthermore, MDA5 knockout rescues an ADAR1 editing-deficient phenotype, suggesting that the most relevant physiological function of ADAR1 is to specifically prevent aberrant activation of MDA5-mediated immune sensing^42–44^. This underscores the suggestion that it is imperative to achieve a balance between RNA editing at key sites, while preventing stimulation of the IFN pathway.

In this context, the present study was designed to examine the relationship between immune activation during pregnancy, the mechanism of RNA editing in the fetus, and the long-term emergence of behavioral phenotypes associated with MND in the offspring. In contrast to previous studies in adults that detected minor or no differences in RNA editing levels between healthy and MND brains^45,46^, our results indicate that activation of the IFN pathway by dsRNA at a critical time in pregnancy leads to changes in gene expression as well as a vast global increase in RNA editing levels, which may produce a high mutation load in the developing fetus.

The study also revealed the presence of tight regulation of coding editing sites during mouse brain development. The deviation from normal editing levels detected in the PolyI:C-treated mice may affect protein function^37,47–49^ and therefore could potentially explain the observed alterations in brain development. As robust activation of ADAR may lead to errant editing at unknown sites, we hypothesize that MIA induces temporary changes in RNA editing patterns during a critical period of (prenatal) brain development. These can be responsible for long-term deleterious effects on brain function in later life, even if they are not measurable by the time the behaviors are observed.

## Results

### Behavioral deficits in PolyI:C-treated mice

In order to verify that indeed the mice we treated prenatally with PolyI:C showed MND-related behaviors, we conducted two behavioral experiments, the prepulse inhibition (PPI) and locomotor response to amphetamine tests. These are considered to be behavioral endophenotypes relevant for schizophrenia^17,18^. The control group had eight females and five males while the PolyI:C group had seven females and seven males.

**Fig. 1:**
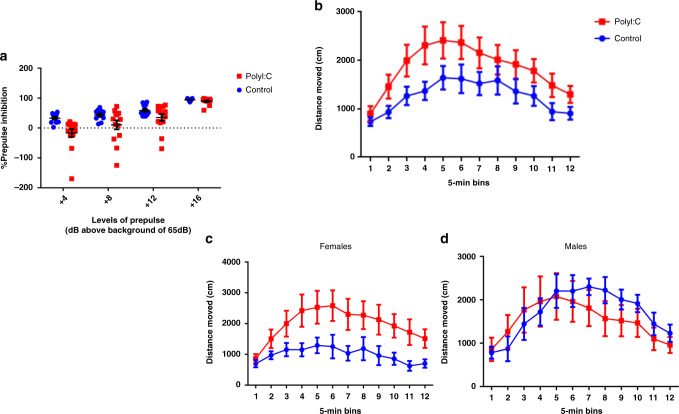
Behavioral changes in the offspring of PolyI:C-treated mothers. **a** PPI data (mean ± SEM) shows the percent of prepulse inhibition of the startle response following the presentation of prepulse-plus-pulse acoustic stimuli. PolyI:C offspring showed a significant PPI deficiency (*n* = 13 Control and *n* = 14 for PolyI:C, two-sided *p* value = 0.002; obtained from *F* test with (2,26) degrees of freedom) compared to controls at four different prepulse intensities (69, 73, 77, and 81 dB), regardless of gender. **b** Locomotor activity measured following an initial 30-min habituation period, a 30-min period following an injection of saline, and a 1-h period following a challenge injection of 2.5 mg/kg amphetamine (data only shown for last segment). PolyI:C mice displayed enhanced locomotor activity compared to control mice (*n* = 13 for Control and *n* = 14 for PolyI:C, two-sided *p* value = 0.02; obtained from *F* test with (1,26) degrees of freedom), and the effect was stronger in females than in males (**c** females: *n* = 8 for Control, *n* = 7 for PolyI:C, two-sided *p* value = 0.03; obtained from *F* test with (1,13) degrees of freedom; **d** males: *n* = 5 for Control, *n* = 7 for PolyI:C, two-sided *p* value = 0.74; obtained from *F* test with (1,11) degrees of freedom). All values are means ± SEM. Source data are provided as a Source Data file.

*PPI*: We assessed sensorimotor gating by the paradigm of PPI of the acoustic startle response, since reduced PPI has been proposed as a biomarker of schizophrenia^50^. In the PPI test, a weak prepulse stimulus suppresses the response to a subsequent startling pulse stimulus. Our PolyI:C-treated mice demonstrated a deficit in PPI, irrespective of gender (Fig. 1a). A 2 × 2 × 4 ANOVA test yielded a highly significant effect of levels (*F* = 75.84, DF = 3/69, *p* < 0.001) indicating that the level of percent PPI increased as the level of prepulse increased. The factor of gender and its interactions were not significant (*F* = 0.82, DF = 1/23, *p* = 0.37). However, the factor of treatment was highly significant in its own right (*F* = 13.46, DF = 1/23, *p* < 0.01), as well as the interaction with prepulse levels. Similar results were observed in gender-specific strata, with slightly less significance, probably due to smaller sample sizes, leading to lower statistical power (Supplementary Fig. 3).

*Locomotor response to amphetamine*: An enhanced reaction to amphetamine is indicative of a dopaminergic imbalance that is considered a schizophrenia endophenotype. The effect of PolyI:C immune challenge was further evaluated by investigation of the sensitivity to the locomotor-enhancing effects of the indirect dopamine- receptor agonist, amphetamine. The sensitivity to acute drug administration was measured by assessing the effects of the drugs on locomotor activity in an open field.

Prenatal PolyI:C exposure did not significantly affect spontaneous locomotor activity in terms of distance traveled in the whole open-field area (Supplementary Fig. 4a). Similarly, the locomotor response to vehicle (saline) treatment was highly comparable between the experimental groups (Supplementary Fig. 4b), and no significant group differences were detected under these conditions.

The systemic administration of amphetamine (2.5 mg/kg, i.p.) resulted in a general increase in locomotor activity. This response was enhanced in PolyI:C offspring (Fig. 1b), as revealed by a 2 × 2 × 12 ANOVA test (*F* = 5.38, DF = 1/23, *p* < 0.03), with the between-subject effects of sex and prenatal treatment as well as 12 5-min time intervals, demonstrating a significant effect of treatment.

A significant three-way interaction of sex × treatment × time intervals (*F* = 2.56, DF = 11/253, *p* < 0.005) as can be seen in Fig. 1c reflects the fact that the enhancement of activity due to amphetamine was much more noticeable in female animals than in the males. In fact, as can be seen in Fig. 1c, d, the female treated animals responded much more to amphetamine than the male treated animals, whereas in the control group, the opposite trend emerged and the male controls responded more than the female controls to the injection of amphetamine.

### MIA drives transcriptomic changes in neuronal gene pathways

**Fig. 2:**
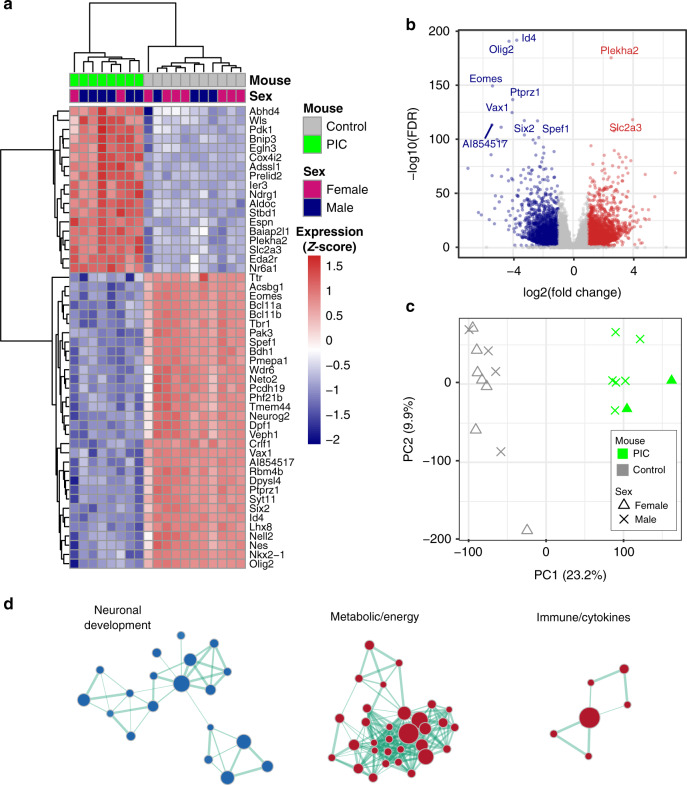
Transcriptomic dysregulation in the PolyI:C mice model. **a** Heat map of the 50 most differentially expressed genes (i.e. smallest FDRs) using normalized (separate *Z*-score computed per gene) TPM expression values. **b** Volcano plot representing the expression changes of all genes. Significantly down- and upregulated genes (log_2_ FC|>1 and FDR < 0.05) are colored navy and red, respectively. Genes that do not show significant expression changes are colored gray. Top 10 most differential genes (i.e. smallest FDRs) are labeled with gene names. **c** PCA analysis. Principal components were computed for each sample using gene expression. The first two principal components (PC1 and PC2) are shown. The percent of variance explained by each component is reported in parentheses. **d** Gene set enrichment analysis performed on gene ontology (GO) terms, identified down- and upregulated gene sets. Each node represents one gene set (i.e. a GO term) where the node size indicates the number of genes in the gene set and the color indicates down- (blue) or up- (red) regulation. In summary, the data nodes are clustered based on the number of shared genes between any two gene sets (using the Enrichment map plugin on Cytoscape—see Methods). Three biological modules emerge: (I) neuronal development, (II) metabolic/energy, and (III) immunological. The distances between the nodes and edge thickness indicate the number of shared genes (closer and thicker indicate more shared genes). The following are the largest gene sets in each module: *GO FOREBRAIN DEVELOPMENT*, *GO NUCLEOBASE CONTAINING SMALL MOLECULE METABOLIC PROCESS*, and GO *REGULATION OF CYTOKINE PRODUCTION* for modules I, II, and III respectively. A full list of the gene sets included in this plot are presented in Supplementary Data 3. Source data are provided as a Source Data file.

After establishing that it was possible to detect MND behavioral deficits in the MIA model, we sought to investigate the transcriptomic effect of immune over-activation, in the offspring fetal brains on the day after the MIA. To this end, we sequenced the brains of 11 control (4 males and 7 females) fetuses and 8 fetuses (6 males and 2 females) from PolyI:C-treated mice at GD10 (see “Methods”). The results were analyzed for differential gene expression using thresholds of |log_2_FC | >1 and false discovery rate (FDR) <0.05, and revealed extensive changes in transcription (Fig. 2), including 2384 upregulated and 1626 downregulated genes (Fig. 2 and Supplementary Data 1). Principal component analysis (see Methods) separated treated from control samples along the first principal component (PC1). In contrast, no gender-based separation was detected (Fig. 2c). Gene Set Enrichment Analysis (GSEA) across gene ontology (GO)^51–53^ terms revealed three main dysregulated clusters in the PolyI:C model, which were related to: [I] brain and neuronal development, [II] energy and metabolism, and [III] immunological signatures—all of which have been described previously in schizophrenia (Fig. 2d and see “Methods”). A full list of up- and downregulated gene sets is presented in Supplementary Data 2.

### Enhanced A-to-I RNA editing in PolyI:C-treated mice

In addition to gene expression analysis, we evaluated the global levels of A-to-I RNA editing directly from transcriptomic data using the *Alu* Editing Index (AEI) method^54,55^, which provides information about the general level of editing in intronic *Alu*. Instead of human *Alu*, we supplied the AEI with mouse genomic Short Interspersed Nuclear Element (SINE) regions, B1, where most of the editing activity in mouse occurs^56^. This level can be compared across samples to give an indication of RNA editing activity, even in cases where only low sequencing coverage is available^55^.

**Fig. 3:**
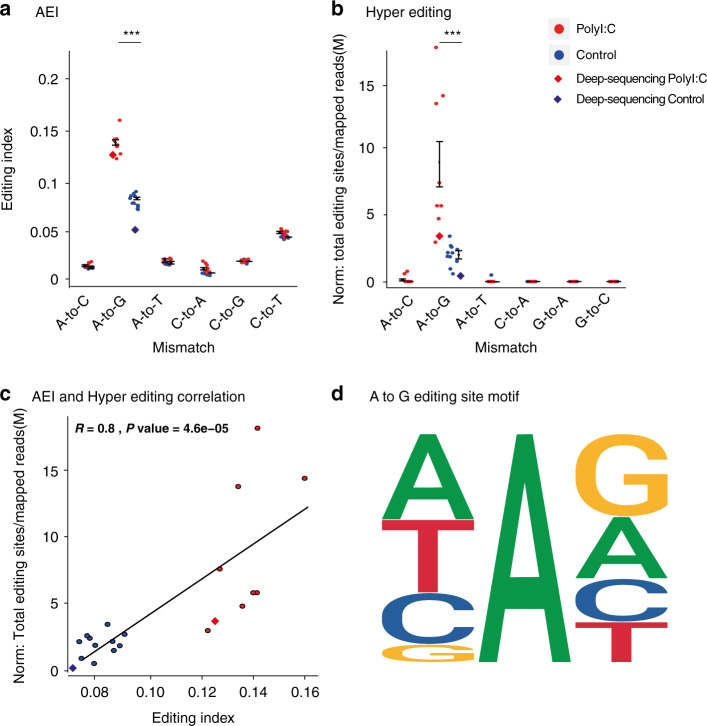
Increased global levels of A-to-I RNA editing in PolyI:C-treated mice. Adenosine to inosine, which is read as guanosine (A2G Editing Index), RNA editing levels in PolyI:C mice sequenced on embryonic day 10 analyzed by **a** Alu Editing Index (AEI) on mouse B1 repetitive elements, and **b** hyperediting (HE) scheme applied to reads with an extensive editing rate that could not be aligned to the reference genome (two-sided Wilcoxon rank-sum test, *p* value = 2.6e−05 (*W* = 88) and 5.29e−05 (*W* = 87), respectively). Other mismatch types are considered noise. The levels of editing from the deep sequencing samples are indicated with a rhombus. **c** AEI and HE normalized editing levels are strongly correlated (Spearman, *R* = 0.8, *p* value = 4.6e−6). **d** The familiar ADAR motif signature for the A-to-G sites could be identified. Source data are provided as a Source Data file. All values are means ± SEM.

The AEI in all B1 regions revealed a significant elevation in global editing in PolyI:C-treated mice compared to that of controls (Fig. 3a). Since PolyI:C treatment activates an IFN response, we hypothesized that the p150 isoform of ADAR1 would be a major contributor to the increased levels of editing detected. An additional AEI analysis of 3472 B1 regions located exclusively within 3′ or 5′ UTRs produced the same significant increase, confirming that the p150 isoform of ADAR1 is responsible for much of the increased editing levels (Supplementary Fig. 5).

In order to verify these findings, we used a hyperediting scheme^57^, as an additional method to identify clusters of editing sites that are overlooked by standard alignment methods (see “Methods”). Here too, the results revealed a significant elevation of global editing in PolyI:C-treated mice compared to controls (Fig. 3b).

The normalized number of editing sites (see “Methods”) identified in the hyperediting analysis correlated well with the AEI in each sample (Fig. 3c)

In order to further analyze the hyperediting output in each group we combined the A-to-G sites. When a site appeared in both the control and the PolyI:C treatment groups, it was classified as a “common site”.

Intersecting the sites in each group with the refseq genes table, we could conclude that the total number of unique editing sites inside genes in the PolyI:C-treated group was 3 times the number of sites in the control group (Supplementary Data 4). Most of these sites were located within introns.

Since the behavioral testing (Fig. 1c, d) indicated differences between male and female offspring after MIA, we also wished to examine the effect of gender on RNA editing. Although the low number of females in the PolyI:C group was insufficient to achieve statistical significance, our results did not indicate sex as a differentiating factor of the levels of RNA editing.

In support of this result, there was no difference in the AEI of the four male and seven female control mice (Supplementary Fig. 6).

To study the possible influence of sex on RNA editing in healthy subjects, we expanded our analysis and included ~330 RNA-seq brain samples from healthy humans, downloaded from The Genotype-Tissue Expression (GTEx) portal (https://gtexportal.org/home/). We calculated the AEI in 138 (93 males, 45 females), 115 (80 males, 35 females), and 80 (54 males, 26 females) brain samples originated from the Cerebellum, Frontal Cortex, and Amygdala, respectively. Here too, no differences in editing levels could be associated with gender in any of the datasets (Supplementary Fig. 7).

To further validate our editing analysis, we estimated the frequency of other types of common transcriptomic mismatch in the regions, since these can often be a result of technical and biological artifacts (noise) rather than bona fide editing. The editing index expected to be the second highest after A-to-G is C-to-T, which represents both a common genomic mutation and the possibility of C-to-U RNA editing, and is therefore useful to estimate the signal-to-noise ratio.

As expected, an A-to-G mismatch was found to be the most frequent editing occurrence in both the AEI and the hyperediting analyses, with a high signal-to-noise ratio when compared to other transcriptomic mismatches. As duplicate reads were removed from all samples before the analysis (see “Methods”), we could verify the effect on editing changes by analyzing the original data. A comparison of the editing in PolyI:C and control mice yielded the exact same statistical power (Supplementary Fig. 8). However, the average signal-to-noise ratio was higher after removal of duplicate reads (average ratio of 2.10, with duplicate reads and 2.30 after duplicate read removal), confirming the removal efficiency. In addition, the known ADAR motif signature was observed around the A-to-G sites^40,58^ (Fig. 3d).

Our analysis was based on a sequencing coverage of 30M reads per sample. To further confirm our results, we repeated the sequencing for two of the samples (one control and one PolyI:C), with 220M reads per sample, and calculated the editing levels. A similar increase in levels of editing was found in both the original and the deep sequencing samples. Specifically, AEI (Fig. 3a) and hyperediting analyses (Fig. 3b) in PolyI:C-treated mice revealed an increase in editing compared to the levels of control. These results reinforce our original findings. Finally, the list of unique editing sites from the hyperediting analysis that overlap refseq genes was twice as high after deep sequencing and with a similar ratio of three times more unique sites in the PolyI:C group than in the control animals (Supplementary Data 4).

As the next step, we used specific primers to measure the expression of ADAR1-p110 and ADAR1-p150 by quantitative real-time PCR (qRT-PCR) (see “Methods”). The sort of viral infection, simulated by PolyI:C, activates an IFN response, which via an IFN-inducible promoter, can lead to overexpression of the ADAR1-p150 isoform and subsequently to increased levels of RNA editing. In this case, it was important to examine whether the PolyI:C treatment indeed caused increased expression of the IFN-inducible ADAR1 isoform. As already discussed, the ADAR1 isoforms have different expression patterns, with ADAR-p110 located exclusively in the nucleus while ADAR-p150 is present both in the nucleus and in the cytoplasm^21^. Since most of the changes in editing occur in introns located in the nucleus, it was important to ascertain that the changes in editing we detected originate from ADAR-p150 activity. The results indicated that PolyI:C exposure markedly increased mRNA expression of *ADAR1-p150*, the IFN induced isoform^20^, with no significant alterations in the expression levels of *ADAR1*-*p110* isoform detected (Supplementary Fig. 9).

Taken together, these results verify the A-to-I RNA editing origin of the observed A-to-G mismatches.

### Altered RNA editing in recoding sites

RNA editing sites in coding sequences represent only a small, albeit important, fraction of the editing activity. We used a set of 59 known, evolutionarily conserved, editing sites^59^ to assess RNA editing level changes in coding sequences. Although millions of years of evolution separate humans and mice, these sites were found to be conserved within the mammalian lineage, and are thus presumed to have an important biological function^60^.

**Fig. 4:**
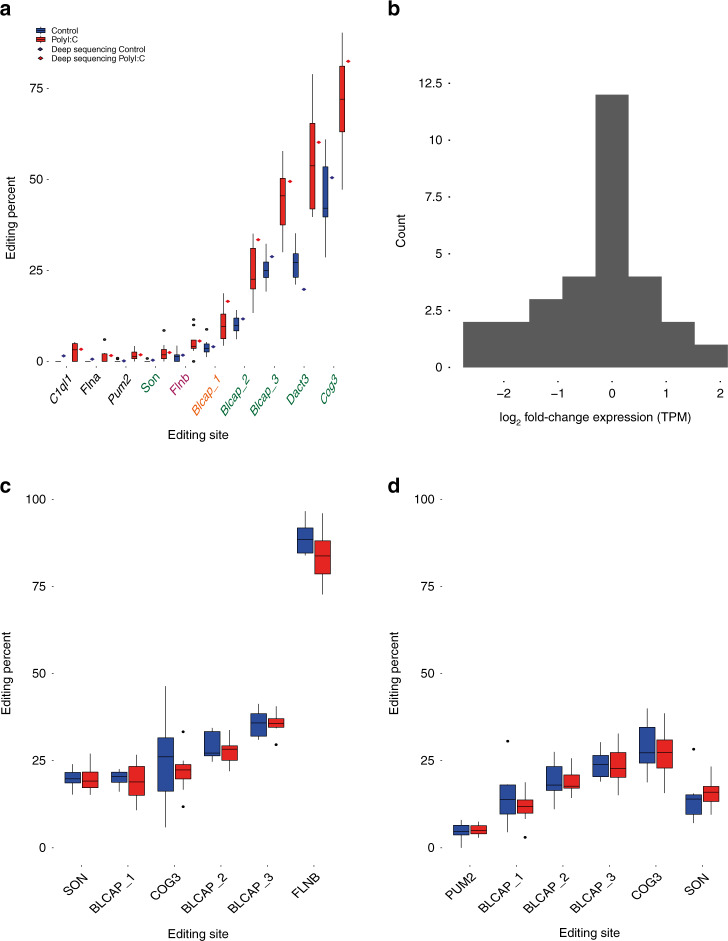
Increased levels of A-to-I RNA editing in recoding sites in PolyI:C mice. **a** RNA editing levels were measured in a set of 59 evolutionarily conserved coding editing sites. Only sites with at least 10 reads sequencing coverage were considered reliable. Ten sites that satisfied the condition exhibited a significant increase in editing levels in the PolyI:C group (*n* = 8) compared to controls (*n* = 11) (two-sided wilcoxon rank test followed by the Benjamini–Hochberg false discovery rate (FDR) multiple testing (0.1)). For each site, editing levels of deep sequenced samples (see “Methods”) are also indicated. The ADAR type^41^ responsible for the editing site is indicated as orange for ADAR1 specific, green for shared ADAR1 and ADAR2, and pink for ADAR2. The levels of editing obtained from the deep sequencing samples are indicated with a rhombus by each site. **b** Log_2_ fold change of gene expression levels (PolyI:C/control) for 30 genes (TPM > 1) harboring the 59 conserved editing sites (average log_2_ fold change = −0.28). **c** Editing level analysis on the 59 conserved editing sites for older PolyI:C (*n* = 9) and control (*n* = 8) mice. The plotted sites are those that overlapped with the differentially expressed sites detected. Represented samples are the frontal cortex of mice subjected to MIA treatment at GD12.5 and sequenced at PD189. **d** Amygdala samples from mice subjected to MIA treatment at GD9 (*n* = 10) and control (*n* = 10) and sequenced at 12 weeks of age. All distributions are presented as box-and-whisker plots (center line, median; box limits correspond to the first and third quartiles; whiskers, 1.5× interquartile range and points, outliers). Source data are provided as a Source Data file.

Many of the editing sites are situated in genes with low levels of expression during development and reliable editing levels could not be determined. However, ten conserved specific editing sites that passed the reads coverage cutoff (see “Methods”) were all found to have significantly increased levels of RNA editing in the PolyI:C-treated group, particularly the sites in Dact3, Cog3, and Blcap genes (Fig. 4a). Notably, Flna, Flnb, Son, Pum2, and C1ql1 also showed subtly increased levels of editing. The highest average change in RNA editing rates was found in the Dact3 editing site (chr7:16885347), where an amino acid is recoded from arginine to glycine. In this case, the editing levels were twice as high in the PolyI:C-treated group, with 52% editing, compared to the controls with only 26% editing. In the Cog3 editing site (chr14:75719719), which recodes from isoleucine to valine, we detected an increase from 45% editing in the control to 68% in the PolyI:C-treated mice.

There were only minimal variations in the gene expression of the genes harboring the 59 conserved editing sites when comparing the PolyI:C and control groups, indicating that differential editing was unlikely to be secondary to alterations in gene expression (Fig. 4b).

Further analysis of the deep sequenced samples showed a similar increase in editing levels in coding sites (Fig. 4a). This analysis revealed additional editing sites in four positions of GRIA2, including a Q/R site that became detectable only in the deep sequencing experiment (Supplementary Table 2).

To highlight the contribution of ADAR1 to the differentially edited sites, we classified each site by the relevant ADAR^41^. Six out of the seven differentially edited sites were substrates for both ADARs (Fig. 4a), thus supporting our claim of the importance of this form of the enzyme.

To broaden our perspective on changes in editing in older offspring of PolyI:C-treated mice, we analyzed additional RNA-seq datasets: (1) nine PolyI:C and eight control frontal cortex samples from mice subjected to MIA treatment at GD12.5 and sequenced on PD189. (2) 10 PolyI:C and 10 control amygdala samples from mice subjected to MIA treatment at GD9 and sequenced at the age of 12 weeks.

As expected, the datasets exhibited no differences between PolyI:C-treated mice and the controls in either the levels of editing in coding sites (Fig. 4c, d and Supplementary Data 5) or in the global editing index (Supplementary Fig. 10). Taken together, these results highlight the potential influence of temporally increased levels of editing on the development of MND, as the result of changes occurring at a critical period for brain development.

### RNA editing is highly regulated during brain development

**Fig. 5:**
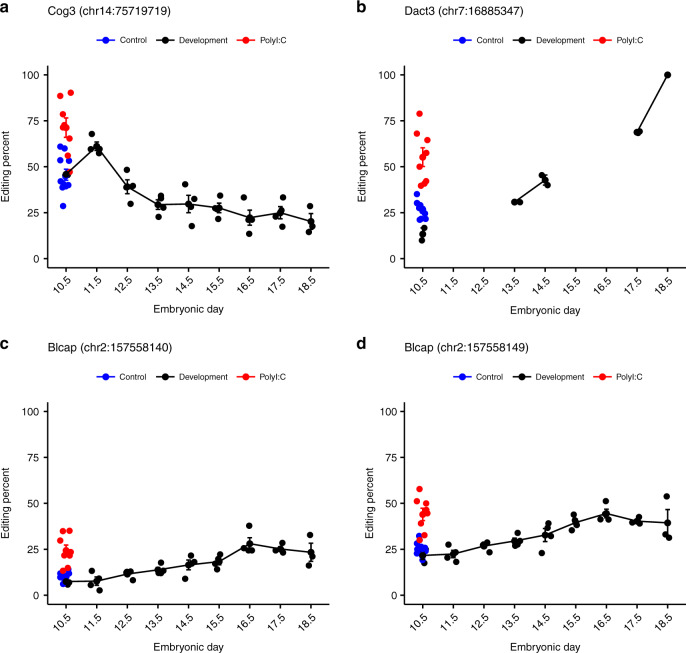
Reproducible pattern of RNA editing levels in embryonic mouse brain development. A-to-I RNA editing levels in evolutionary conserved coding editing sites in the brains of healthy mouse embryos during brain development (*n* = 4 for days 11.5–17.5, *n* = 3 for days 10.5 and 18.5). Levels of RNA editing from PolyI:C (*n* = 8) and control (*n* = 11) mice from the MIA model are indicated on GD10 when the brains were sequenced. The top four recoding editing sites that showed the largest change in editing levels in the MIA model are **a** COG3 (chr14:75719719), **b** DACT3 (chr7:16885347), **c** BLCAP (chr2:157558110), and **d** BLCAP (chr2:157558140). Editing level is indicated only when sufficiently expressed (see “Methods”). All values are means ± SEM. Source data are provided as a Source Data file.

RNA editing modification has been found to play a critical role in various biological conditions and diseases. To examine the typical level of variations in RNA editing levels during normal development, we analyzed^61^ three to four samples of fetal mouse brain at every developmental day, from embryonic day 10–18. The results were used to track the changes in A-to-I RNA editing levels in conserved coding sites^59^ and to calculate the AEI. This analysis revealed that the RNA editing levels^62^ of the various coding sites over the tested period of embryonic development were highly reproducible between samples (Fig. 5 and Supplementary Figs. 11 and 12). This stability highlights the tight regulatory network that operates during fetal development. Most of the analyzed sites exhibited a general trend of elevation in editing levels as development progressed^38,63,64^. However, certain sites exhibited a peak in editing levels on a specific day. For example, COG3 editing was moderately reduced over the period analyzed but displayed a peak in editing levels on embryonic day 11. Taken together, these results emphasize the importance of accuracy and repeatability of RNA editing levels during brain development, while a deviation from normal levels may harm the developmental process.

## Discussion

MNDs such as schizophrenia are complex diseases, and arise from the synergistic effects of genetics and environmental factors. Studies in recent years have identified a large number of risk loci associated with schizophrenia^65^. Here, we provide material evidence for the presence of an additional risk factor in the form of alternative genetic information, namely RNA. We propose that changes in RNA content and expression during a certain sensitive period in embryonic development, following an environmental insult giving rise to in utero MIA, can influence the delicate neurodevelopmental process, and contribute to the risk of MND onset.

Using the well-established MIA model of risk for the occurrence of MND, we could demonstrate that the well characterized long-term MIA-induced behavioral changes (reduced PPI and increased amphetamine sensitivity) are associated with distinct transcriptomic changes in the prenatal period. RNA analysis of fetal brains in the period following the MIA led to several compelling insights and revealed extensive dysregulation in gene sets related to brain and neuronal development. To our knowledge, this is the first time that RNA-seq has been performed during this time period of mouse development following MIA, as most gene expression studies have considered only the adult offspring of MIA^66–68^. Since fetal brain tissue at this stage is scant, our results provide information at the level of gene enrichment. Nonetheless, they strongly implicate the changes noted in the future development of MND such as schizophrenia, by identifying defects in brain and neuron development.

A recent study by Breen et al.^69^ revealed changes in RNA editing in coding sites in cortical samples from adult individuals with schizophrenia. Since PolyI:C is an inducer of IFN, which promotes ADAR1-p150 expression^20^, it was of interest to consider whether changes in A-to-I RNA editing also play a role in the development of this disease. As predicted, we identified extensive changes in A-to-I RNA editing following MIA, with large differences in global editing levels between the PolyI:C-treated group and the control group, where the offspring of PolyI:C-treated mice exhibited much higher levels of editing overall.

The A-to-I editing changes were investigated further by examining changes at specific editing sites, specifically a set of 59 editing sites in coding sequences known to be conserved in mammalian evolution^59^. Even though most of the functional changes caused by this editing are not yet known, the conservation of these sites over tens of millions of years speaks to their importance. RNA editing in conserved sites usually leads to amino acid substitutions. Here, we detected a general increase in editing in the mice treated with PolyI:C, and particularly in the recoding sites within the DACT3, COG3, and BLCAP genes. These are particularly interesting in that the Dact3 gene is essential for normal brain development, and important in embryonic development of the central nervous system^70^, while COG3, a part of the COG complex, interacts with Biogenesis of Lysosome-Related Organelles Complex 1 (BLOC-1), which contains the schizophrenia susceptibility factor dysbindin. Dysbindin expression was previously shown to be reduced in the hippocampi and cortical areas of schizophrenia patients, which implicates the protein in molecular pathways leading to schizophrenia^71–75^.

Since sequencing depth is crucial to confirm editing levels, we repeated the experiment on one sample from each group, with a much deeper coverage of 220M reads. Importantly, not only did the alterations in RNA editing become more robust, but the deep sequencing also revealed additional differentially edited sites in coding sequences. These included a Q/R GRIA2 site, which is a crucial site for editing in humans and mice^76^. Deviations in editing at the GRIA2 gene can lead to severe disease phenotypes^77,78^. Moreover, editing levels of GRIA2 were previously reported to be altered in postmortem brains from schizophrenia patients^79,80^. Analysis of hyperediting, which identifies clusters of editing sites that are overlooked by standard alignment, led us to conclude that there were significantly more editing sites inside genes in the PolyI:C group compared to control. While most of these sites were situated inside introns and therefore do not alter functionality, they may have an effect by changing the biogenesis of circRNA, alter the splicing pattern of the pre-mRNA or otherwise influence the expression of mRNA by editing the miRNA binding sites^81^.

Since all these changes occur at the level of a single cell, the overall effect may be slight and difficult to detect. However, if they take place at a critical time in development, they may be enough to cause a meaningful impact. It is important to remember that these changes are temporary and may be undetectable when the RNA or protein composition of an adult brain is analyzed.

Indeed, additional transcriptomic analysis of older MIA mice revealed no difference between PolyI:C-treated mice and controls in either the global editing index, or in the levels of editing in coding sites (Supplementary Fig. 10 and Supplementary Data 5), indicating that the changes we observed at 24 h post MIA are indeed transient. To validate this point in humans, we performed further analysis on autism and schizophrenia postmortem brain datasets^82–84^, and detected no significant differences in overall editing levels compared to control samples (Supplementary Figs. 13 and 14). Notable recent studies have reported site-specific changes in RNA editing in human adult patients with schizophrenia^69^ and autism^45^. Our new analysis of coding editing sites in adult NMD patients likewise indicated changes in editing levels in several coding sites (Supplementary Data 6 and 7). This may represent an opening for further future research.

In order to better understand the implications of our results, we performed an analysis of RNA editing throughout the development of a healthy mouse brain. Most compellingly, the developmental transcriptomic data revealed the developmental process to be tightly regulated with strong reproducibility in AEI across samples at the same stage. This evidence of regulation was confirmed when we calculated the editing levels in conserved known edited coding sequences on each day. Interestingly, comparing the results obtained on gestational day 11 to those from our MIA model experiment revealed similar levels of editing in the control groups and an elevation in the PolyI:C group. In light of the observation that A-to-I editing is so tightly regulated during development, it is reasonable to suppose that any deviation from normal levels may severely affect brain development. A possible scenario for the MIA-induced hyperediting is that in addition to necessary and routine editing, the overexpression of ADAR1 in response to infection and the consequent up-regulation in IFN may give rise to undesirable extra editing. This could take the form of editing at novel sites simply due to the greater availability of the editing enzyme. If these sites are located in a coding sequence and alter a significant protein, in a significant cell, at a significant time point for brain development, the resulting changes could eventually lead to a phenotypic change.

While our results introduce a new potentially influential factor in the development of MND, they should be viewed in light of certain limitations. First, we extracted the RNA at a single time point of 24 h after MIA induction. Since we are the first to sequence and analyze this time point in the establishment of the disease, there is no possible confirmation from the literature. Future studies should repeat the experiment with various time points in order to track and further characterize the effect. In addition, in order to prove that the association between changes in RNA editing of prenatally and long-term behavioral changes is indeed causal, it will be necessary to follow the mice longitudinally. Lastly, studies utilizing MIA models have previously reported sex differences^85–87^ and indeed, we observed sex differences in the amphetamine-challenge behavioral test. Gender-related behavioral characteristics have also been noted both in human schizophrenia patients and mice models^88^. This divergence in behavior between sexes cannot be explained by differences in RNA editing nor by changes in gene expression at 24 h post MIA, as analysis of both yielded no indication of gender-related effects (Fig. 2c and Supplementary Figs. 6 and 7).

In conclusion, we demonstrate here, for the first time, a molecular mechanism that links MIA to long-term behavioral deficits relevant for MND, such as schizophrenia. We describe immense gene expression changes, which occurred following MIA particularly in three main major dysregulated clusters related to brain and neuronal development, energy and metabolism, and immunological signatures—all of which have been implicated previously in schizophrenia pathobiology. Further analysis of brain developmental data revealed a precise regulation of RNA editing levels in coding sequences required for normal brain development. We therefore offer evidence to suggest that A-to-I RNA editing may be considered a contributing factor linking MIA to the onset of MND. While changes in editing may not be observable in adult patients with schizophrenia, our results in a mice model may suggest that major global and regional changes that occur at the embryonic stage have long-term significance to brain development. These changes may cause transient, and in most cases, untraceable brain changes, which may still lead to disease development, even if they are no longer in evidence by the time disease symptoms manifest.

## Methods

### Animals

Naive female and male C57BL6/J mice, between 8 and 12 weeks of age, were obtained from Envigo (Israel). Litter mates of the same sex were kept in groups of three to five mice, and were maintained in standard conditions of constant temperature (22 ± 1 °C), humidity (relative, 40%), and on an ad libitum food and water diet under a 12:12 light–dark cycle. All experimental protocols were authorized by the Tel Aviv University Committee of Animal Use for Research and Education.

Breeding began after 7 days of acclimatization to the new animal holding room. Groups of 2–3 females were moved to separate cages with some urine-soiled wood chip bedding from a cage containing male C57BL6/J mice. On the fourth day of exposure to male urine, females were exposed to one male and allowed to mate. The next morning, the male mouse was removed from the cage, and the females were weighed. This day was referred to as gestational day (GD) 0. Females who showed an increase of over 2 g in body weight after 9 days were considered pregnant.

We used two cohorts of offspring: (1) mice that were prenatally sacrificed soon after the MIA procedure to allow an examination of the short-term effects of MIA on RNA editing patterns in the brain (PolyI:C = 8, Control = 11) and (2) mice that were used to study the long-term behavioral effects of prenatal MIA (PolyI:C = 14, Control = 13).

### Prenatal treatment

PolyI:C sodium (Sigma‐Aldrich #P1530, St. Louis, MO, USA) was dissolved in sterile 0.9% saline on the day of injection. Injections were made via the intravenous route into the tail vein under mild physical constraint, and the volume of injection was 5 ml/kg. The animals were returned to the home cage post-injection.

On gestational day 9, pregnant C57BL/6J dams were injected with PolyI:C (5 mg/kg/ml) or vehicle (saline) into the tail vein. The timing of injection was based upon the work of Feldon and Meyer^15^, which suggested that MIA in early pregnancy can lead to behavioral and cognitive dysfunctions relating to schizophrenia in the offspring.

### Evaluation of the short-term effects of MIA on RNA editing patterns in the offspring fetal brain

The group of pregnant mice designated for RNA analysis of the fetuses were killed by decapitation 24 h after PolyI:C or vehicle treatment (i.e., on GD10). The abdominal cavity was exposed, and the uterus was removed and placed in a Petri dish filled with ice-cold phosphate-buffered saline (PBS). The uterus was then transferred to another Petri dish containing ice-cold PBS and dissected under visual guidance using a dissecting microscope.

The separated fetal heads were immediately placed in *RNA save* solution (Biological Industries, Israel) in an Eppendorf tube and frozen immediately by immersion in liquid nitrogen. Samples were stored at −80 °C until RNA extraction.

A comparison of the expression of brain markers^89^ in animals from the control group and healthy brain samples^61^, sequenced on the same developmental day, confirmed that the tissue originated mainly from the brain (Supplementary Fig. 1).

### Evaluation of long-term behavioral effect of MIA in the offspring

For behavioral testing, the offspring born to PolyI:C and vehicle-treated mothers were weaned, and their sex was determined at postnatal day 24. They were housed 3–5/cage, each consisting of male or female offspring derived from multiple independent litters.

Behavioral testing started at 14–16 weeks of age and included both male and female offspring. Animals were gently handled 3–4 times per week in the 2 weeks prior to behavioral testing.

### PPI of the acoustic startle

For the PPI test^16^, A startle chamber (San Diego Instruments, San Diego, CA, USA) was used to measure startle reactivity. The startle chamber comprised a nonrestrictive cylindrical enclosure made of clear Plexiglas attached horizontally on a mobile platform, which in turn rested on a solid base inside a sound-attenuated isolation cubicle. A high-frequency loudspeaker mounted directly above the animal enclosure inside each cubicle produced a continuous background noise of 65 dB (A-scale) and various acoustic stimuli in the form of white noise.

For PPI of the acoustic startle reflex, subjects were presented with four types of trials. These were pulse-alone trials, prepulse-plus-pulse trials, prepulse-alone trials, and trials in which no discrete stimulus, other than the constant background noise, was presented (denoted here as “no-stimulus” trials). A reduction of the magnitude of the startle after prepulse-plus-pulse trials relative to that obtained after pulse-alone constitutes PPI. The pulse stimulus employed was 120 dBA in intensity and 40 ms in duration. Prepulses of various intensities: 69, 73, 77, 81, and 85 dB, corresponding respectively to 4, 8, 12, 16, and 20 dB above background, were employed. The duration of prepulse stimuli was 20 ms. The SOA (stimulus onset asynchrony) of the prepulse and pulse stimuli in the prepulse-plus-pulse trials was 100 ms.

A session began with the animals being placed in the Plexiglas enclosure and allowed to acclimatize to the apparatus for 2 min before the first trial. The first six trials consisted of startle-alone trials, which served to habituate and stabilize the animals’ startle response. Subsequently, the animals were presented with 12 blocks of discrete test trials. Each block consisted of one trial of each of the following trial types: pulse-alone, prepulse-plus-pulse trials of each of the five levels of prepulse, prepulse-alone of each of the five levels of prepulse, and no stimulus (i.e., background alone). The session was concluded with a final block of six consecutive startle-alone trials. The interval between successive trials was variable with a mean of 15 s (ranging from 10 to 20 s).

%PPI was calculated as ((mean startle response to 120 dB pulse-alone-mean startle response following a prepulse)/mean startle response to 120 dB pulse alone) × 100.

### Acute response to amphetamine

To investigate the behavioral activation induced by an acute amphetamine injection, all mice were tested in an open-field test composed of three sessions. First, spontaneous locomotor activity of the mice in response to novelty was recorded during 30 min without any drug treatment (Habituation Session 1). After this, all animals received an intraperitoneal injection of saline (NaCl 0.9%, 5 ml/kg body weight) and were returned immediately to the open-field arena for a further 30-min session (Saline Session 1). Finally, the locomotor activity response of the mice to an acute amphetamine treatment was recorded for a further 60-min after an intraperitoneal injection of 2.5 mg/kg d-amphetamine (Amphetamine Session 1). Thus, all animals served as their own controls.

### Isolation and purification of RNA

Total RNA was extracted using the RNeasy Mini kit (Qiagen, Dusseldorf, Germany). Isolated and purified total RNA was then quantified using a NanoDrop 2000 Spectrophotometer (Thermo Scientific) and the absorbance ratios *A*_260_:*A*_280_ and *A*_260_:*A*_230_ were measured. The integrity of RNA was evaluated based on RIN acquired via capillary gel electrophoresis performed using Agilent 4200 TapeStation in combination with Agilent RNA ScreenTape System (Agilent Technologies, Santa Clara, CA, US). All RNA samples went through DNase Treatment Kit (Qiagen, Dusseldorf, Germany) before proceeding to the next step.

### PolyA selection and library preparation

For this particular study, we prepared a library of 19 RNA samples using NEBNext RNA ultra II RNA library preparation kit (E7770L). The input amount of total RNA was 200 ng which was in the recommended range. All RNA samples underwent PolyA selection following the manufacturers’ protocols.

Samples were multiplexed using suitable molecular barcodes and resulting cDNA pools were processed according to the NextSeq System Denature and Dilute Libraries Guide. Firstly, the concentration of libraries was measured using a Qubit Fluorometer in combination with the Qubit dsDNA HS Assay Kit (Invitrogen, Carlsbad, CA, USA). Secondly, libraries were analyzed by Agilent 4200 TapeStation in combination with Agilent High Sensitivity D1000 ScreenTape System (Agilent Technologies, Santa Clara, CA, US) according to the manufacturer’s protocol.

### Next-generation sequencing

Paired-read sequencing of the libraries with a read length of 150 was performed with NextSeq 500 Sequencing System using NextSeq 500/550 High Output v2 kit (20024906 Illumina). PhiX Control v3 (Illumina) was added at 1% to all pools as an internal control before sequencing.

### Quantitative real-time PCR (qRT-PCR)

Two hundred and fifty nanograms of RNA were used in a reverse transcription reaction using the Verso cDNA Synthesis Kit (Thermo Fisher Scientific, Cat. No. AB 1453). The cDNA obtained was used in real‐time PCR. Platinum SYBR GreenqPCR SuperMix-UDG w/ROX (Invitrogen, Carlsbad, CA, USA) was used to quantify gene expression. The number of mice used for this experiment was *n* = 7 for the control mice and *n* = 5 for the PolyI:C group, since not enough DNA was available from this group. Specific primers were used to amplify ADAR-p110 and ADAR-p150, and β-actin was included in each experiment as a loading control. The fold change in expression was determined using the ΔCt method. The following primer sequences were used for PCR—ADAR-p110 Forward Primer (FP): 5′-GCAGCGTCCGAGGAATCG-3′, Reverse Primer (RP): 5′-TAAGACTCCGGCCCCTGTG-3′; ADAR-p150 FP: 5′-CACTATGTCTCAAGGGTTCAGGG-3′, ADAR-p150 RP: 5′-CACTTGCTATGCTCATGACTAGGG-3′; and β-actin FP: 5′-AGAGCATAGCCCTCGTAGAT-3′, β-actin RP: 5′-CCCAGAGCAAGAGAGGTATC-3′.

### Data preprocessing and quality control

In RNA-seq data, duplicate reads bias, defined as reads that are identical and on the same strand, can occur and may affect the accuracy of analysis. These reads can be produced by the PCR cycles conducted before sequencing. To reduce sequencing biases, duplicate reads were removed using the PrinseqLite script version 0.20.4 (http://prinseq.sourceforge.net/). An average of 7% of the reads were removed from each sample (min 3.74%, max 11.02%).

Transcriptomic data quality was evaluated using the FastQC quality control tool version 0.10.1 (ref. ^90^).

The reads were mapped to the mouse reference genome version GRCm38/mm10 using STAR aligner version 2.5.2b^91^. To ensure perfect alignment, we used a non-permissive parameter of only 5% possible mismatches for each read (--outFilterMatchNminOverLread 0.95).

### Global RNA editing levels in SINE repetitive elements

The most highly edited mobile element in the mouse genome is B1 (ref. ^56^). To measure the editing in *B1* elements, we used the AEI method version 1.0 (refs. ^54,55^).

Briefly, we averaged the number of A-to-G mismatches across all B1 adenosines weighted by the total coverage at B1 adenosine positions. The higher the index, the more global editing activity occurs in a sample.

### Hyperediting

An additional approach to RNA-editing evaluation used was a hyperediting scheme^57,92^. Heavily edited reads will not align to the reference genome due to the high load of mismatches. These reads serve as input to the hyperediting pipeline. The pipeline transforms all As and Gs in both the unmapped RNA sequences and the reference genome. The transformed reads are then realigned and editing levels can be evaluated.

To minimize the effect of the library size, we used the STAR aligner to normalize the total editing sites in each sample by the number of reads that were uniquely mapped.

### RNA editing in individual recoding sites

Site-specific RNA editing levels for sites known to be edited in coding sequences were calculated using REDIToolKnown version 1.0.4, which is part of the REDItools package^28^. The list of editing sites consists of 59 sites which are conserved between human and mice^59^.

We used parameters that allow a minimum one read supporting the variation (−v 1), minimum 0.001 editing frequency (−n 0.001), exploring one base near the splice junction (−r 1), minimum one read coverage, and trimming of five bases at both ends of the reads (−T 5-5).

For reliable editing evaluation, we removed sites which had less than 10 reads of alignment coverage. We also removed sites where less than 50% of the group’s samples showed editing signals. The statistical significance of differences between two groups was tested using the Wilcoxon rank-sum test with default parameters, followed by the Benjamini–Hochberg FDR multiple testing (0.1).

### Differential expression and gene set enrichment analysis

Gene expression levels in units of transcripts per million (TPM) were computed using the Salmon tool (version 0.11.2)^93^ following “tximport” R library version 1.12.3 (ref. ^94^) with default parameters and mouse genome version GRCm38/mm10. Principal components were computed for each sample using log_2_ (TPM + 0.1) of all genes. The prcomp R function was used. Differential gene expression was evaluated on Salmon transcript counts using DESeq^95^. For GSEA^96,97^ genes were transformed into their human homolog and ranked by multiplication of *p* value by log_2_ fold change for each gene. Mouse genes corresponding to more than one human gene were included multiple times with the same value. The pre-ranked option was used with the GSEA software version 4.0.2. Clustering of gene sets was performed in Cytoscape (version 3.4)^98^ using the *Enrichment map* module with default values. Clusters with >5 nodes (i.e. gene sets) were included in the plot.

### Sex determination of the mice

The sex of each mouse fetus was determined by analyzing the gene expression levels of chromosome Y-linked genes DDX3Y and UTY. A threshold of expression levels in TPM units <1 was set to define female mice (Supplementary Fig. 2). Supplementary Table 1 lists the sex of each sample.

### Statistics and figures

The statistical calculations on the data and figure drawing were made using Rstudio, an integrated development environment for R (http://www.rstudio.com/).

For behavioral testing, to account for the experimental design with two factors (treatment and noise level or time bins), unequal sample sizes in the treatment factor, and same mouse exposed to multiple noise levels or measured on several time bins (repeated measures), we used linear mixed models with random mouse effects, and sex, treatment group, time interval/noise level as fixed effects, with distance/%PPI as outcome, followed by analysis of variance. Statistical analysis was performed using the publicly available R package lme4. Datasets, code, and reproducible reports of statistical analyses of the behavioral experiments are provided in Supplementary Notes 1–4.

### Reporting summary

Further information on research design is available in the Nature Research Reporting Summary linked to this article.

## Supplementary information

Supplementary Information

Description of Additional Supplementary Files

Supplementary Data 1

Supplementary Data 2

Supplementary Data 3

Supplementary Data 4

Supplementary Data 5

Supplementary Data 6

Supplementary Data 7

Reporting Summary

## Data Availability

RNA-seq data of PolyI:C-treated mice and controls were generated in this study and have been deposited in the SRA (BioProject ID: PRJNA602886). Additional RNA-seq datasets of PolyI:C-treated mice were downloaded from the SRA database (ERP014069, SRP221742). RNA-seq datasets of schizophrenic human brain were obtained from the SRA database (SRP102186, SRP073813). RNA-seq dataset of ASD human brain was obtained from PsychENCODE portal (syn4587615). RNA-seq datasets of healthy human brain were obtained from the GTEx project via dbGaP (phs000424.v8.p2). RNA-seq dataset of fetal mouse brain development was downloaded from the ArrayExpress Archive (E-MTAB-6798). All data and Supplementary information are provided with the paper. Additional information is available upon reasonable request to the corresponding authors. Source data are provided with this paper.

## Source data

Source Data
