# Supplementary material for: Increased RNA editing in maternal immune activation model of neurodevelopmental disease
Source: Nat Commun. 2020 Oct 16;11:5236. doi: 10.1038/s41467-020-19048-6 (PMC7567798; doi:10.1038/s41467-020-19048-6)
Supplement: Supplementary file 1 — Supplementary Information [file 41467_2020_19048_MOESM1_ESM.pdf]

Supplementary Information

Increased RNA Editing in Maternal Immune Activation Model of Neurodevelopmental Disease

Tsivion-Visbord et al.

Supplementary Figures

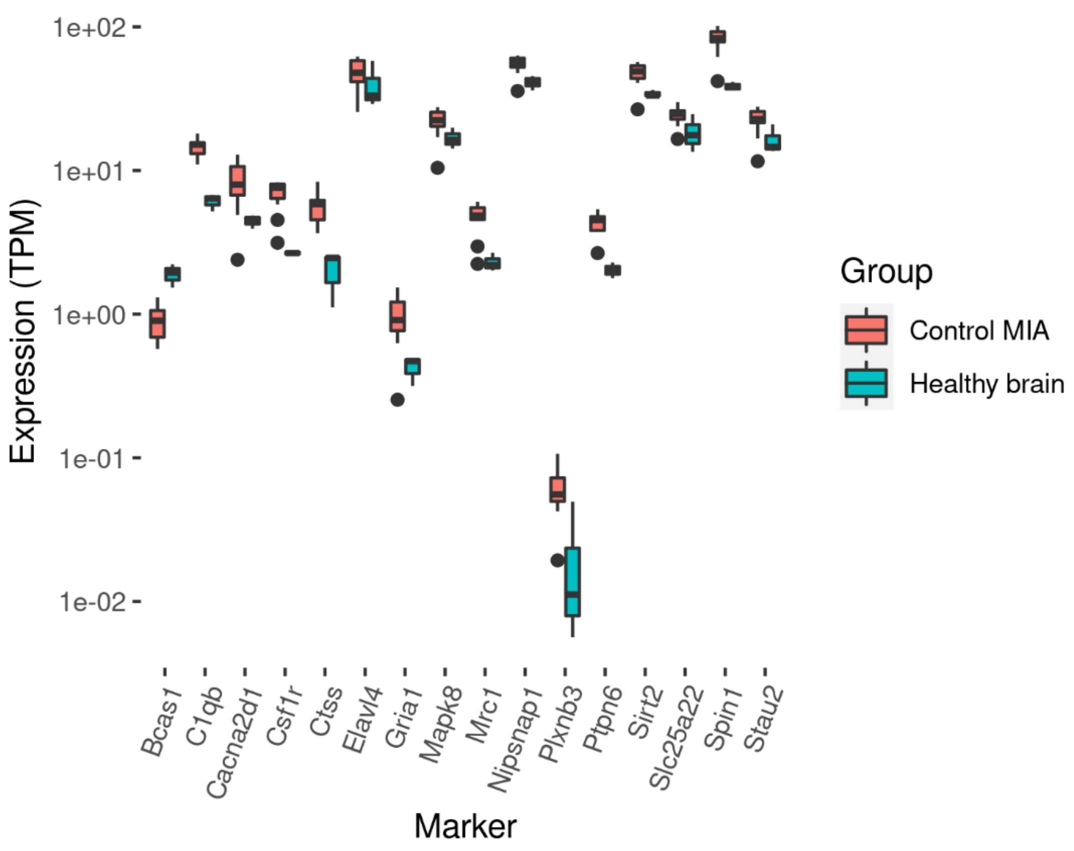

Supplementary Figure 1

Brain tissue of origin verification. A comparison between the gene expression levels of 16 mouse brain markers (Dai et al. 2019) in control samples from our MIA experiment (n = 11), and healthy brain samples (n = 3) from a recently published authoritative resource of gene expression across mouse brain development (Cardoso-Moreira et al. ,Nature, 2019). Both groups were sequenced on gestational day 10. Distributions are presented as box-and-whisker plots (center line, median; box limits correspond to the first and third quartiles; whiskers, 1.5× interquartile range and points, outliers). Source data are provided as a Source Data file.

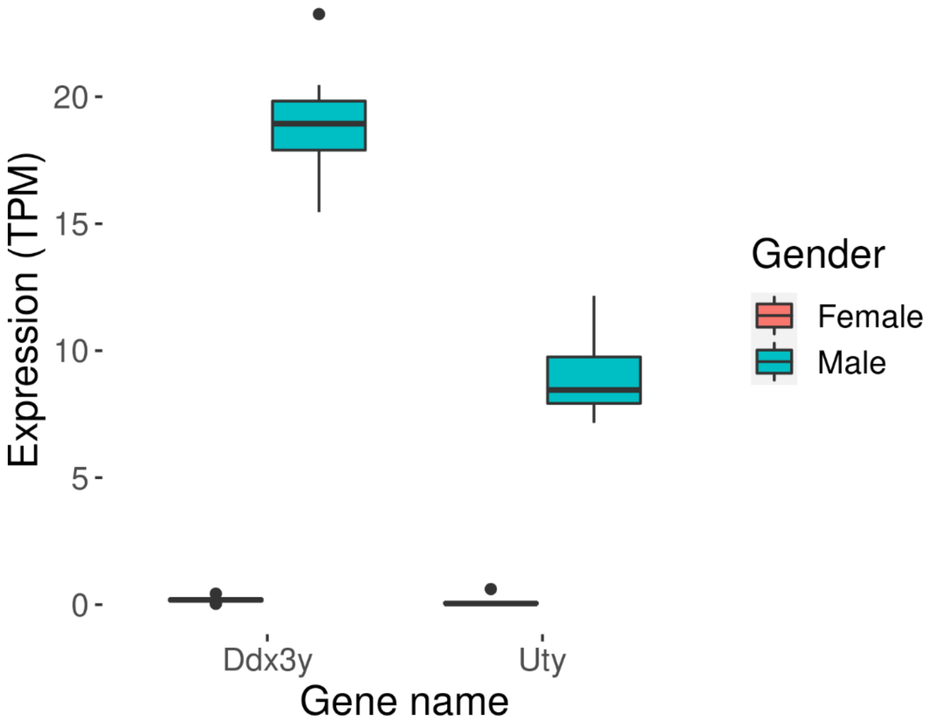

Supplementary Figure 2

Sex determination of the mice. The sex of each mouse fetus was determined by analyzing the gene expression levels of chromosome Y linked genes DDX3Y and UTY (n=10 for male and n=9 for female). A threshold of expression levels in TPM units < 1 was set to define female mice. Distributions are presented as box-and-whisker plots (center line, median; box limits correspond to the first and third quartiles; whiskers, 1.5× interquartile range and points, outliers). Source data are provided as a Source Data file.

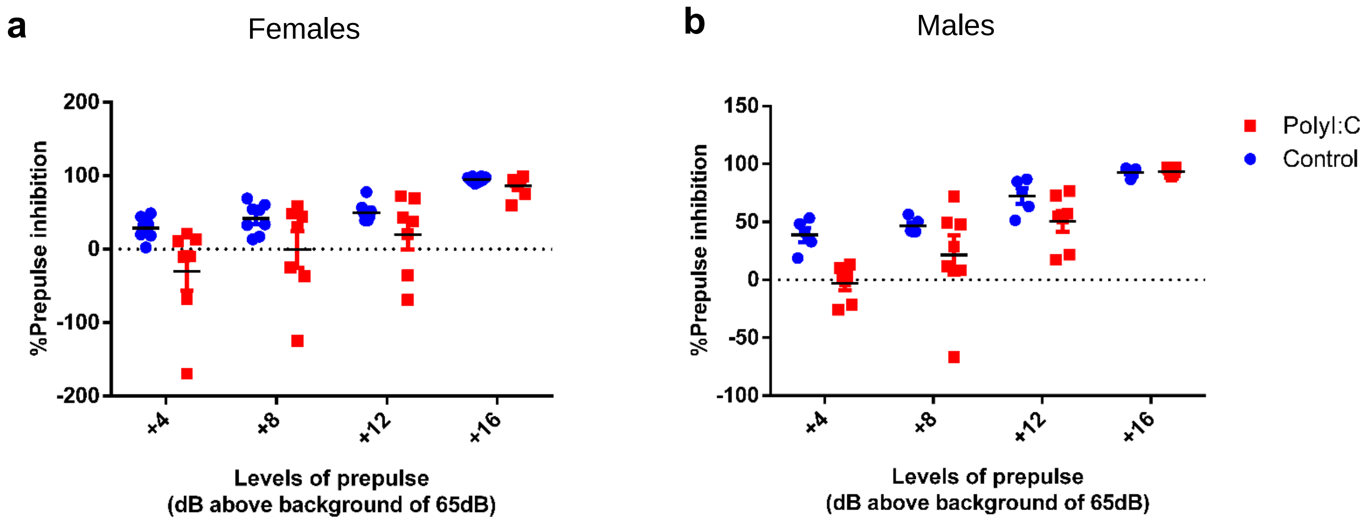

Supplementary Figure 3

Prepulse inhibition test. PPI data (mean ± SEM) shows the percent of prepulse inhibition of the startle response following the presentation of prepulse-plus-pulse acoustic stimuli. Four different prepulse intensities (69, 73, 77 and 81dB) were measured. PolyI:C females (a) showed an almost significant PPI deficiency (n=8 for control, n=7 for PolyI:C, two sided p-value =0.057; obtained from F test with (1,13) degrees of freedom) and Poly I:C males (b) showed a significant deficiency (n=5 for control, n=7 for PolyI:C, two sided p-value =0.01; obtained from F test with (1,10) degrees of freedom). All values are means ± SEM. Source data are provided as a Source Data file.

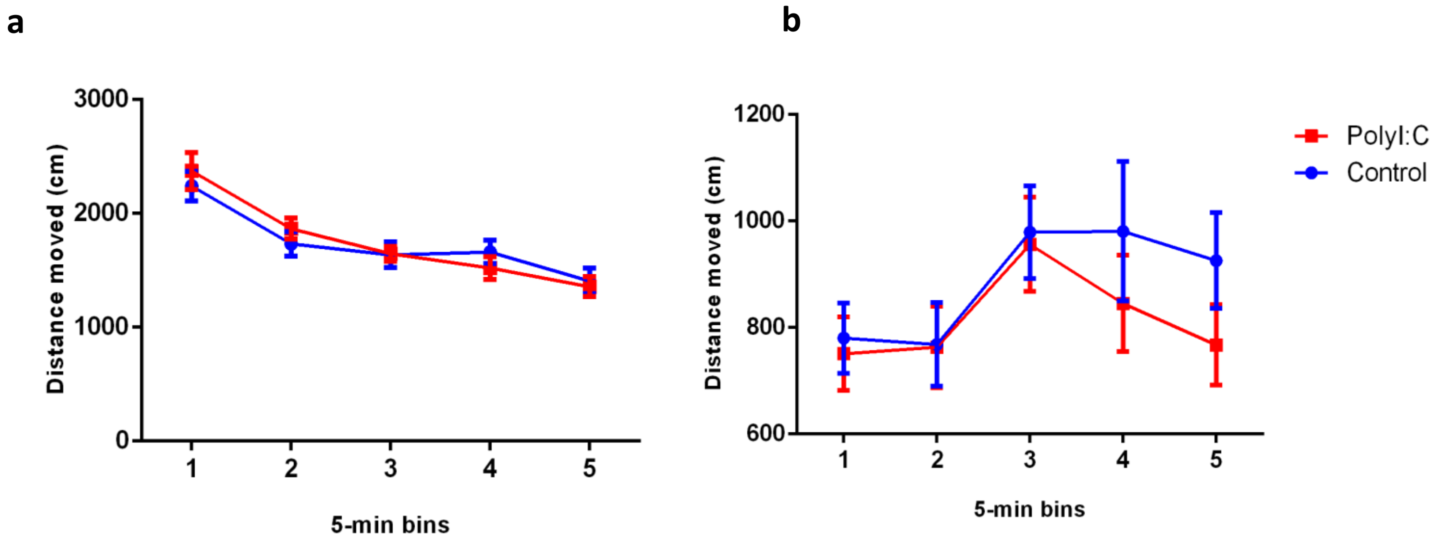

Supplementary Figure 4

Locomotor response to amphetamine. (a) During 30 min acclimation to the open field arena, PolyI:C treated group showed no difference than control, as all groups had a decline in activity after initial reaction, thereby exhibiting acclimation to the open field arena (n=13 for Control, n=14 for PolyI:C, two sided p-value=0.42; obtained from F test with (1,23) degrees of freedom). (b) Reaction to saline injection likewise showed a lack of difference between the groups (n=13 for Control, n=14 for PolyI:C, two-sided p-value =0.42; obtained from F test with (1,23) degrees of freedom). All values are means ± SEM. All values are means ± SEM. Source data are provided as a Source Data file.

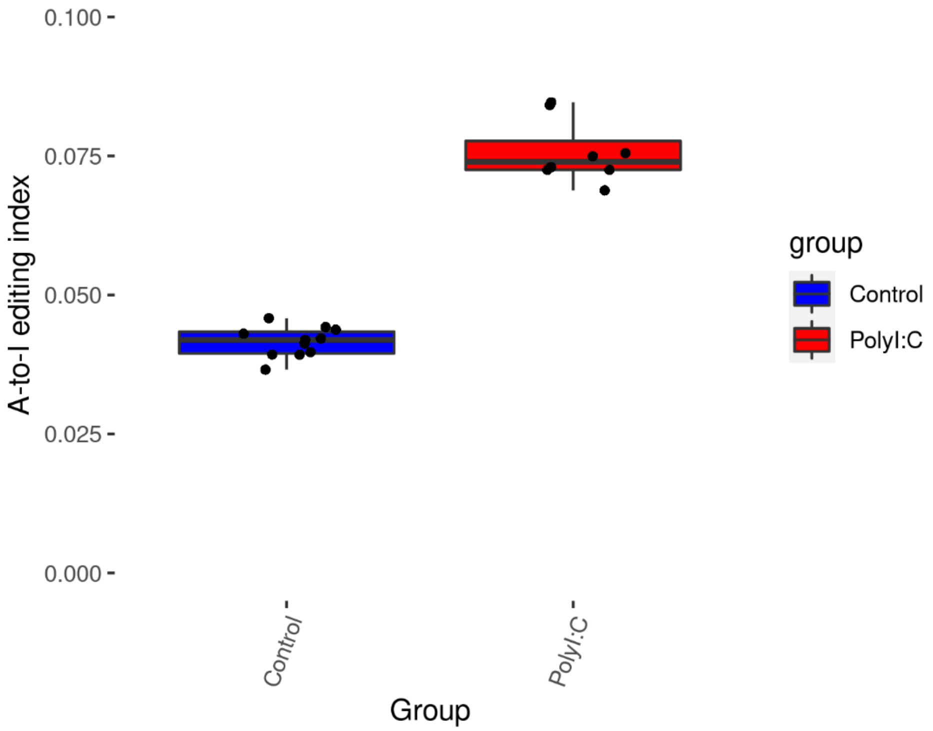

Supplementary Figure 5

ADAR1 p150 isoform contribution to the global increased editing levels. AEI analysis in 3472 B1 element regions located only within 5' and 3' UTR of PolyI:C (n = 8) and control (n = 11) mice (Two-sided wilcoxon rank-sum test, P. Value = 2.64e-05). Distributions are presented as box-and-whisker plots (center line, median; box limits correspond to the first and third quartiles; whiskers, 1.5× interquartile range and points, outliers). Source data are provided as a Source Data file.

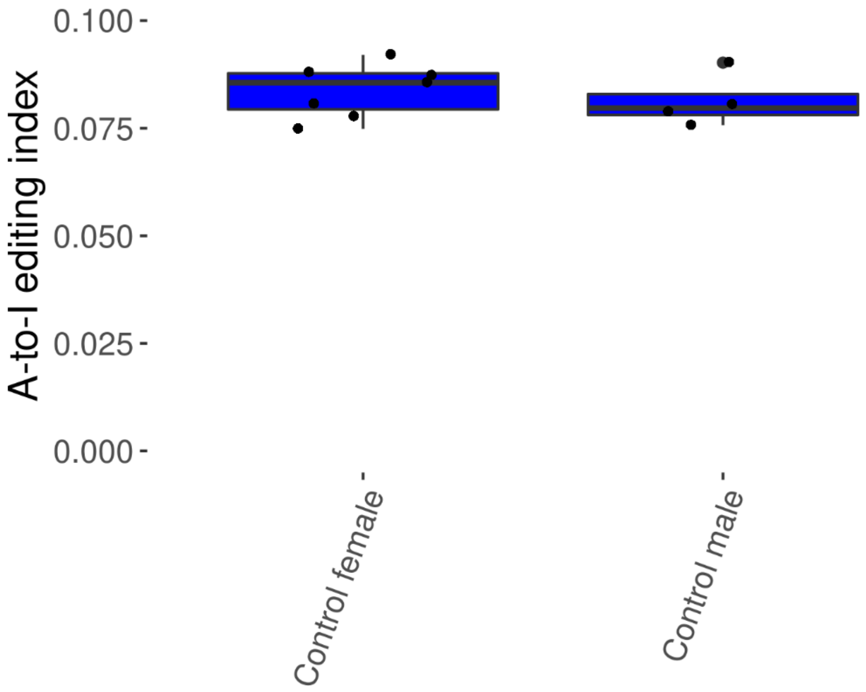

Supplementary Figure 6

Sex as a differentiating factor of the levels of RNA editing in MIA control mice. AEI of male (n = 4) and female (n =7) control mice comparison (Two-sided wilcoxon rank-sum test, P. value = 0.65). Distributions are presented as box-and-whisker plots (center line, median; box limits correspond to the first and third quartiles; whiskers, 1.5× interquartile range and points, outliers). Source data are provided as a Source Data file.

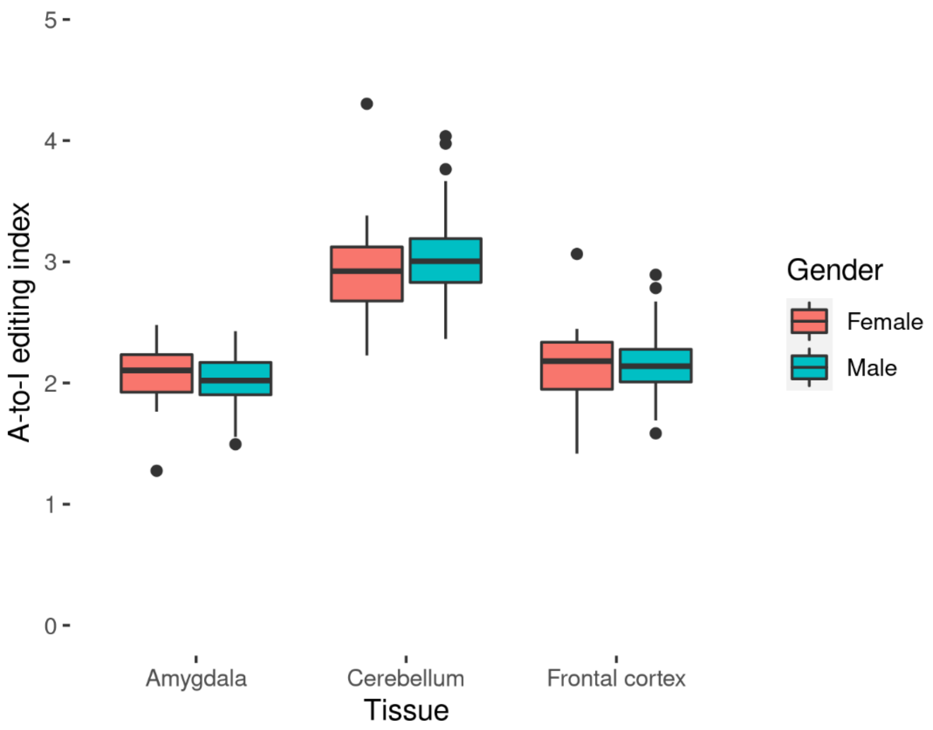

Supplementary Figure 7

Sex as a differentiating factor of the levels of RNA editing on GTEx healthy brain samples. AEI analysis of RNA-seq brain samples from healthy humans (n = 333), downloaded from The Genotype-Tissue Expression (GTEx) portal (https://gtexportal.org/home/). We calculated the AEI of brain samples originated from the Cerebellum (n = 138; 93 males and 45 females), Frontal Cortex (n = 115; 80 males and 35 females) and Amygdala (n = 80; 54 males and 26 females) (Two-sided wilcoxon rank-sum test, P. value = Cerebellum: 0.12, Frontal Cortex: 0.74, Amygdala:0.32). Distributions are presented as box-and-whisker plots (center line, median; box limits correspond to the first and third quartiles; whiskers, 1.5× interquartile range and points, outliers). Source data are provided as a Source Data file.

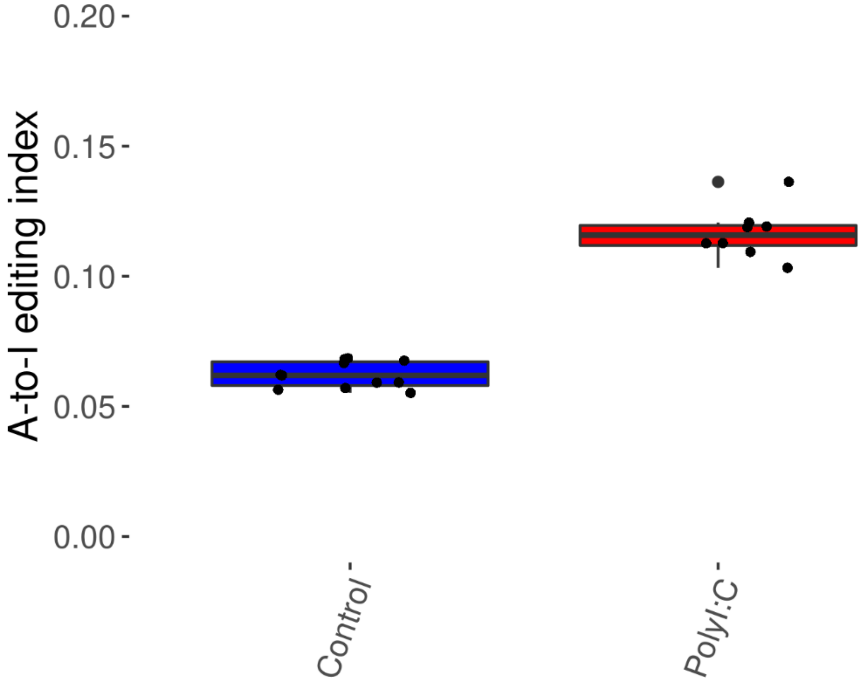

Supplementary Figure 8

Global editing levels in the original data before the removal of duplicate reads. AEI analysis of PolyI:C (n = 8) and control (n = 11) mice (Two-sided wilcoxon rank-sum test, p. value = 2.6e-05). Distributions are presented as box-and-whisker plots (center line, median; box limits correspond to the first and third quartiles; whiskers, 1.5× interquartile range and points, outliers). Source data are provided as a Source Data file.

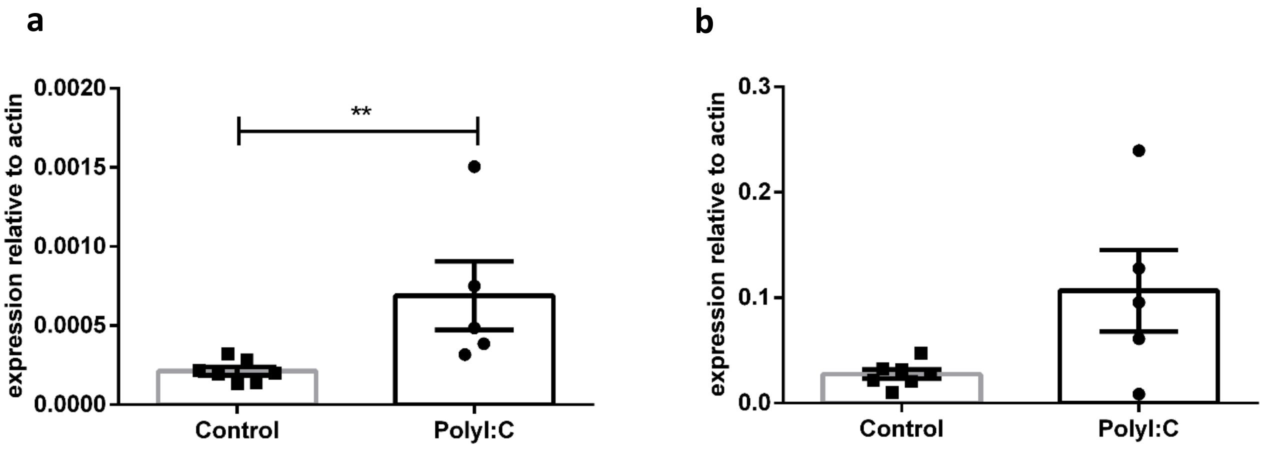

Supplementary Figure 9

mRNA expression of ADAR1 isoforms as detected by qRT-PCR, in mice whose mothers were exposed to PolyI:C and in control mice. PolyI:C injection of the pregnant mice stimulates ADAR1-p150 (a) and ADAR1-p110 (b) expression in the brains of the fetuses. Pregnant mice were injected intravenously with PolyI:C (5mg/kg), and were killed 24 hours after the treatment, at which point the fetus's heads were obtained. mRNA expression of indicated genes was measured with the SYBR Green real-time PCR using their specific primers. Bars represent mean±SEM. Values of fold mRNA expression of ADAR1-p150 and ADAR1-p110 (n=7 for control, n=5 for PolyI:C). Statistical significance of the differences between the groups was assessed with the Mann-Whitney test: **P<0.01. **P<0.01. Source data are provided as a Source Data file.

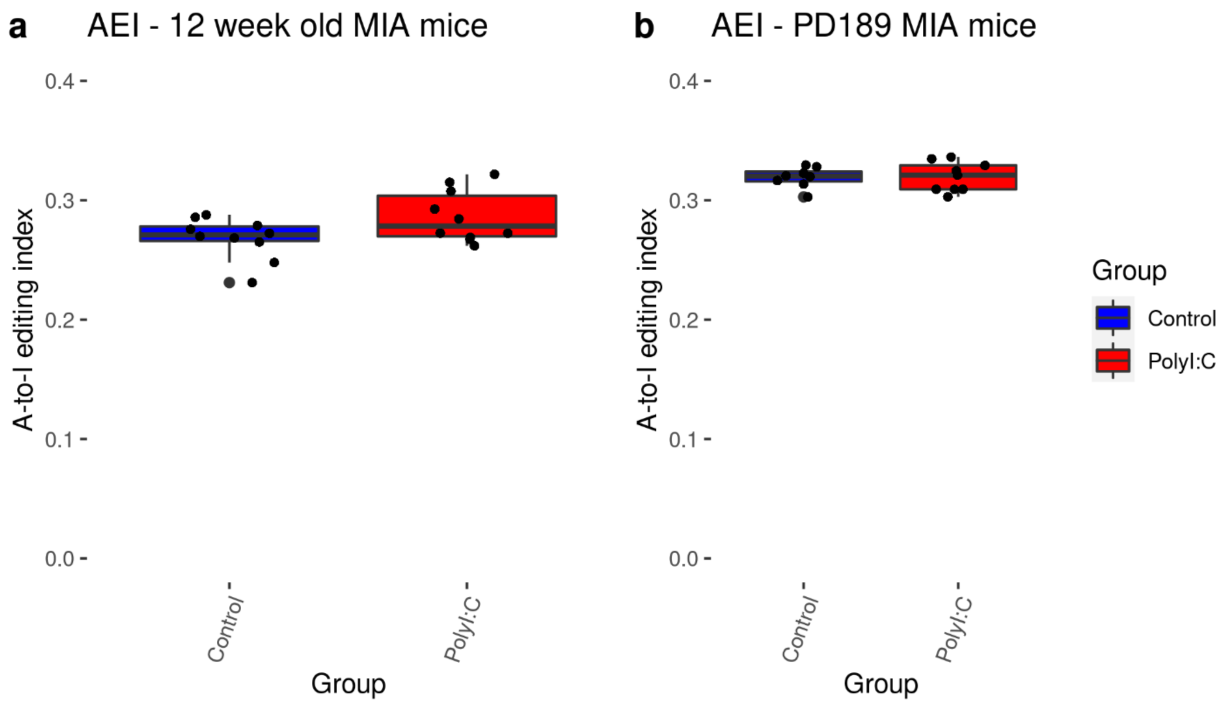

Supplementary Figure 10

Global editing analysis on older PolyI:C and control mice. (a) AEI of frontal cortex samples from mice subjected to MIA treatment at GD12.5 and sequenced at PD189 (Control = 8, PolyI:C = 9) and (b) AEI of amygdala samples from mice subjected to MIA treatment at GD9 and sequenced at 12 weeks of age (Control = 10, PolyI:C = 10). All distributions are presented as box-and-whisker plots (center line, median; box limits correspond to the first and third quartiles; whiskers, 1.5× interquartile range and points, outliers). Source data are provided as a Source Data file.

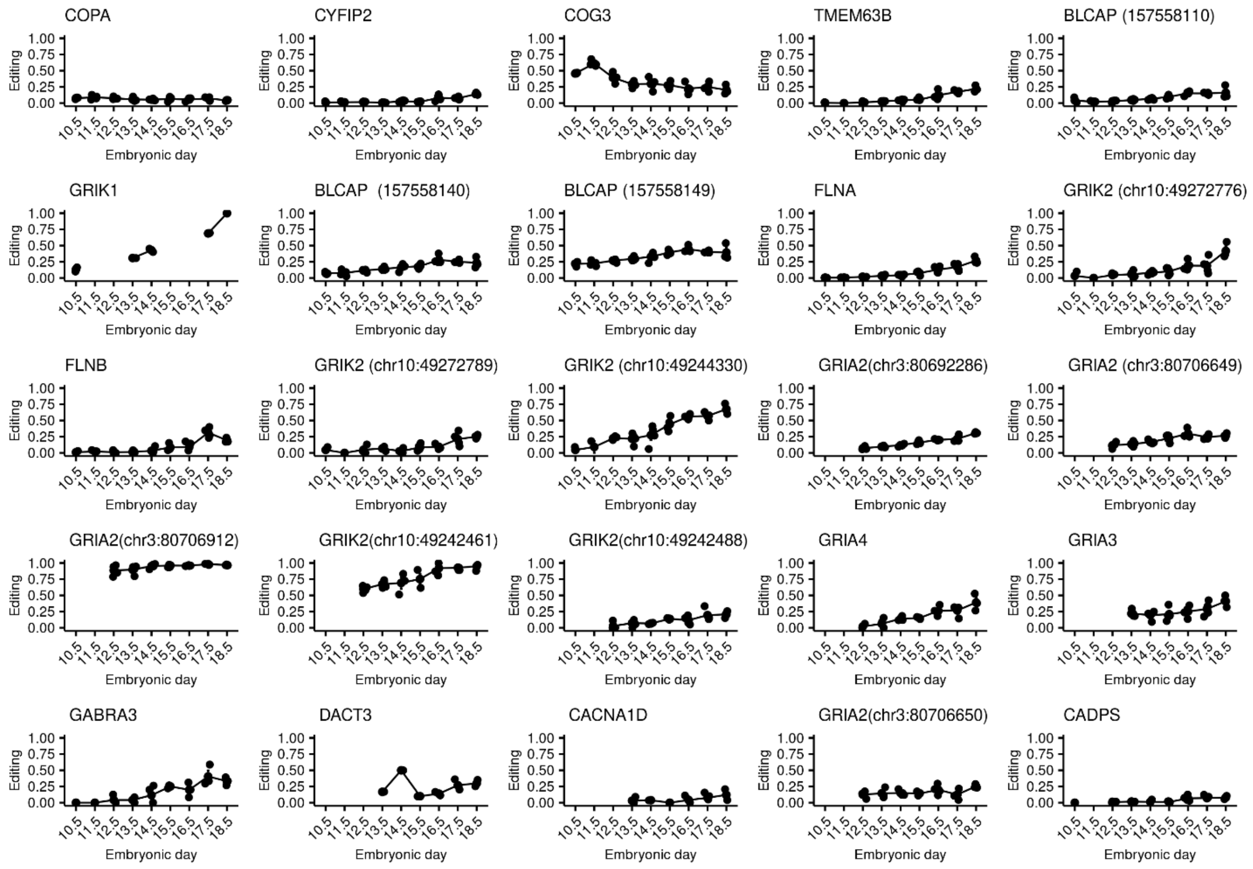

Supplementary Figure 11

Typical level of variations in RNA editing analysis in specific coding sites during normal development. We analyzed the editing levels of fetal mouse brain samples at every developmental day, from embryonic day 10 through 18 (n = 4 for days 11.5 to 17.5, n = 3 for days 10.5 and 18.5). The results were used to track the changes in A-to-I RNA editing levels in conserved coding sites. Most of the analyzed sites exhibited a general trend of elevation in editing levels as development progressed. Source data are provided as a Source Data file.

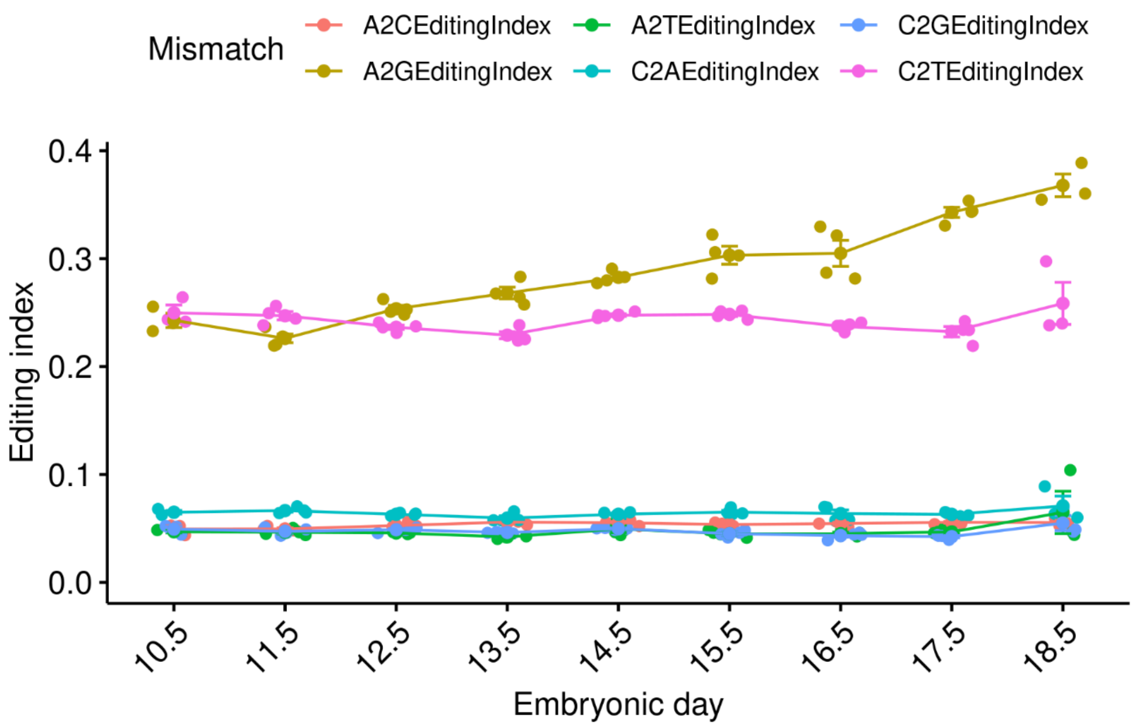

Supplementary Figure 12

Typical level of variations analysis in global RNA editing levels during normal development. We analyzed the AEI of fetal mouse brain at every developmental day, from embryonic day 10 through 18 (n = 4 for days 11.5 to 17.5, n = 3 for days 10.5 and 18.5). All values are means ± SEM. Source data are provided as a Source Data file.

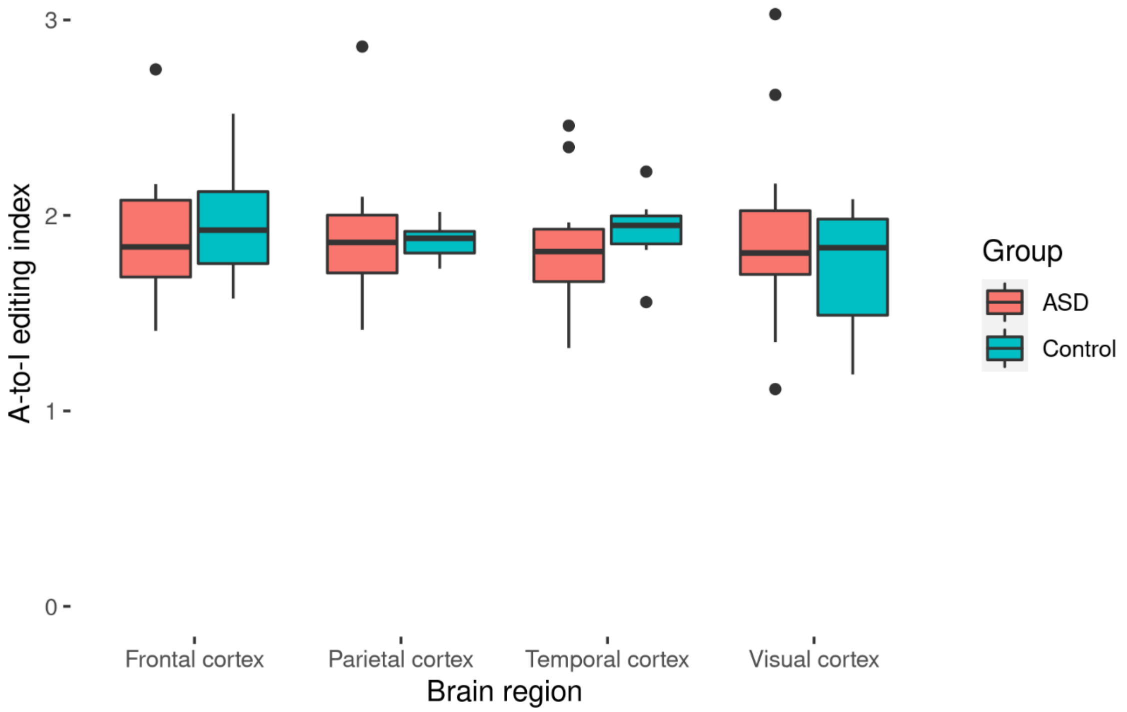

Supplementary Figure 13

AEI analysis on data sets from various regions of postmortem brain from ASD patients (Two-sided wilcoxon rank-sum test, Frontal cortex: Control = 14, ASD = 7, P. value = 0.68; Parietal cortex: Control = 12, ASD = 8, P. value = 0.43; Temporal cortex: Control = 7, ASD = 11, P. value = 0.68; Visual cortex: Control = 8, ASD = 15, P. value = 0.97). Distributions are presented as box-and-whisker plots (center line, median; box limits correspond to the first and third quartiles; whiskers, 1.5× interquartile range and points, outliers). Source data are provided as a Source Data file.

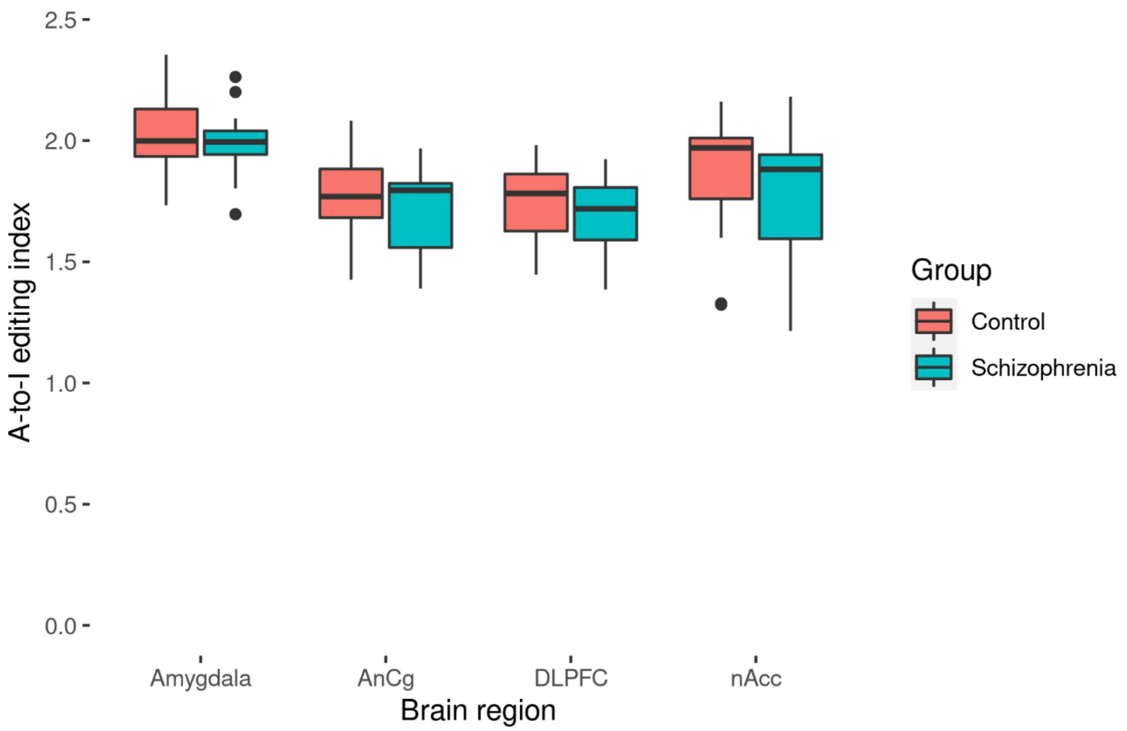

Supplementary Figure 14

AEI analysis on data sets from various of postmortem brain regions from schizophrenia patients (Two-sided wilcoxon rank-sum test, Amygdala: Control = 24, Schizophrenia = 22, P. value = 0.81; Anterior cingulate cortex (AnCg): Control = 30, Schizophrenia = 28, P. value = 0.41; Nucleus accumbens (nAcc): Control = 27, Schizophrenia = 27, P. value =0.21; Dorsolateral prefrontal cortex (DLPFC): Control = 30, Schizophrenia = 29, P. value = 0.08). Distributions are presented as box-and-whisker plots (center line, median; box limits correspond to the first and third quartiles; whiskers, 1.5× interquartile range and points, outliers). Source data are provided as a Source Data file.

Supplementary Table 1. Sex classification for each fetus sample

Sample name	Sex
Con-A1_S17	Female
Con-A2_S12	Male
Con-A3_S19	Male
Con-B1_S11	Male
Con-B2_S8	Female
Con-B3_S13	Female
Con-B4_S3	Female
Con-C1_S7	Male
Con-C2_S6	Female
Con-C3_S10	Female
Con-C4_S2	Female
PIC-A1_S20	Male
PIC-A1_S9	Male
PIC-A2_S16	Female
PIC-A4_S18	Male
PIC-C1_S15	Male
PIC-C2_S14	Female
PIC-C3_S5	Male
PIC-C4_S4	Male

Supplementary Table 2. RNA editing levels in conserved coding sites - deep sequencing analysis.

Region	Position	Strand	Gene name	Site	Control-%editing	PolyI:C-%editing
chr7	16885347	+	DACT3	DACT3	19.8	60.2
chr3	80692286	-	GRIA2	GRIA2_1	10.4	46.2
chr14	75719719	-	COG3	COG3	50.5	82.5
chr3	80706649	-	GRIA2	GRIA2_2	0	25
chr5	77407731	-	IGFBP7	IGFBP7_1	16.7	40.9
chr2	1.58E+08	-	BLCAP	BLCAP_4	38.5	62.3
chr2	1.58E+08	-	BLCAP	BLCAP_2	11.7	33.4
chr2	1.58E+08	-	BLCAP	BLCAP_3	28.7	49.5
chr10	49244330	-	GRIK2	GRIK2_1	5.4	25
chr2	1.58E+08	-	BLCAP	BLCAP_1	4	16.6
chr9	4456006	-	GRIA4	GRIA4	5.7	14.6
chr3	80706912	-	GRIA2	GRIA2_5	83.7	87.9
chr14	7936048	+	FLNB	FLNB	1.6	5.5
chr16	91656133	+	SON	SON_2	0.3	2.3
chr11	46272643	-	CYFIP2	CYFIP2	0	1.9
chr11	1.03E+08	-	C1QL1	C1QL1	1.4	3.2
chr12	8750269	-	PUM2	PUM2	0.1	1.7
chr17	45662949	-	TMEM63B	TMEM63B	0.5	1.9
chr1	1.72E+08	+	COPA	COPA	3.6	5
chrX	74226649	-	FLNA	FLNA_1	0.6	1.7
chrX	74226862	-	FLNA	FLNA_2	0.9	1.9
chr15	38491612	-	AZIN1	AZIN1_1	0	0.3
chr16	91655615	+	SON	SON_1	0.1	0.3
chr14	12411582	-	CADPS	CADPS	0	0
chr17	27502795	-	GRM4	GRM4	0	0
chr14	30066121	-	CACNA1D	CACNA1D	0	0
chr12	46700334	-	NOVA1	NOVA1	0	0
chr10	49272789	-	GRIK2	GRIK2_3	0	0
chr6	1.27E+08	-	KCNA1	KCNA1	0	0
chr15	38491613	-	AZIN1	AZIN1_2	0.1	0
chrX	72445292	-	GABRA3	GABRA3	1.1	0
chrX	41654252	+	GRIA3	GRIA3	20	14
chr3	80706908	-	GRIA2	GRIA2_4	6	0
chr3	80706650	-	GRIA2	GRIA2_3	14.3	7.7
chr10	49272776	-	GRIK2	GRIK2_2	6.8	0
chr5	77407783	-	IGFBP7	IGFBP7_2	18.9	10

Statistical analysis of pre-pulse inhibition in polyI:C treated offspring experiment

Contents

Set data file name and read data 1

Check the number of mice in each group 1

Visualize the mice responses to exposure 2

ANOVA table 3

Female-only analysis 4
ANOVA table 4
Female-specific effect estimates 4

Male-only analysis 4
ANOVA table 4
Male-specific effect estimates 5

Set data file name and read data

```
data_file_name <- "PPI_response_dataset.csv"
dat <- read.csv(data_file_name)
```

Check the number of mice in each group

and whether all mice were assessed at all levels:

```
table(dat[,c("level", "treatment", "sex")])
```

```
## , , sex = female
##
##          treatment
## level     control treatment
##   PPI_69        8         7
##   PPI_73        8         7
##   PPI_77        8         7
##   PPI_81        8         7
##
## , , sex = male
##
##          treatment
## level     control treatment
##   PPI_69        5         7
##   PPI_73        5         7
```

```
##   PPI_77       5         7
##   PPI_81       5         7
```

Visualize the mice responses to exposure

```
dat <- data.table(dat) ## working with data.table is easier
setkeyv(dat, c("treatment", "subj"))
dat$level <- factor(dat$level, levels = unique(dat$level))
p <- ggplot(data=dat, aes(x=level, y=response, group = subj, color = treatment)) +
  geom_bar(aes(x=level, y=response, fill = treatment), stat = "identity", position= "dodge")+
  xlab("Noise level") +
  ylab("Response value")
p + facet_wrap(~sex, ncol = 1)
```

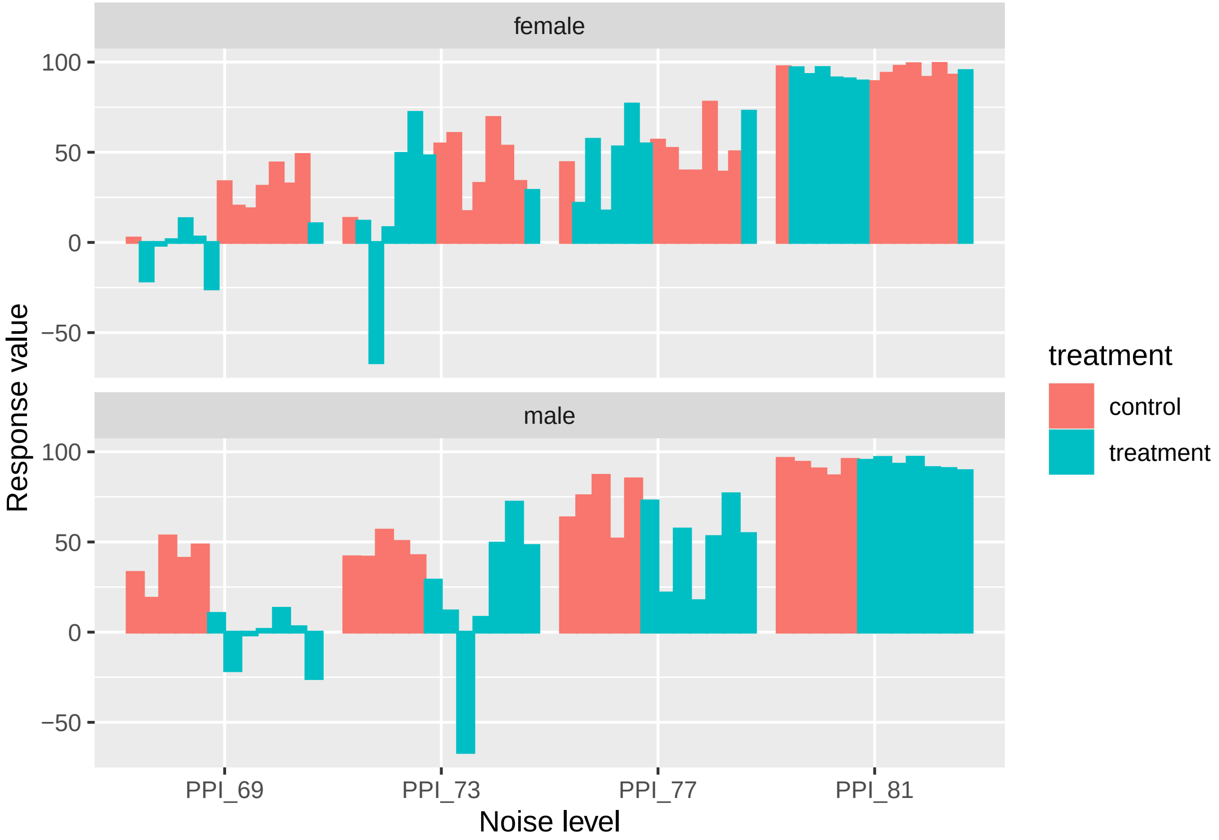

Average across mice in the treatment groups.
Adding bars based on SEs of percent inhibition.

```
dat[,mean_response := mean(response, na.rm = TRUE), by = c("level", "treatment")]
dat[,low_bar := mean(response, na.rm = TRUE) -
        sd(response, na.rm = TRUE)/sqrt(sum(!is.na(response))), by = c("level", "treatment")]
dat[,high_bar := mean(response, na.rm = TRUE) +
      sd(response, na.rm = TRUE)/sqrt(sum(!is.na(response))), by = c("level", "treatment")]
### movement averaged across treatment groups:
p <- ggplot(data=dat, aes(x=level, y=mean_response, group=treatment, color = treatment)) +
  geom_bar(aes(x=level, y=mean_response, fill = treatment), stat = "identity", position= "dodge")+
  geom_errorbar(aes(x = level, ymin = low_bar, ymax = high_bar), position = "dodge", size = 0.5) +
  xlab("Noise level") +
```

```
  ylab("Average response (by treatment group)")
p + facet_wrap(~sex, ncol = 1)
```

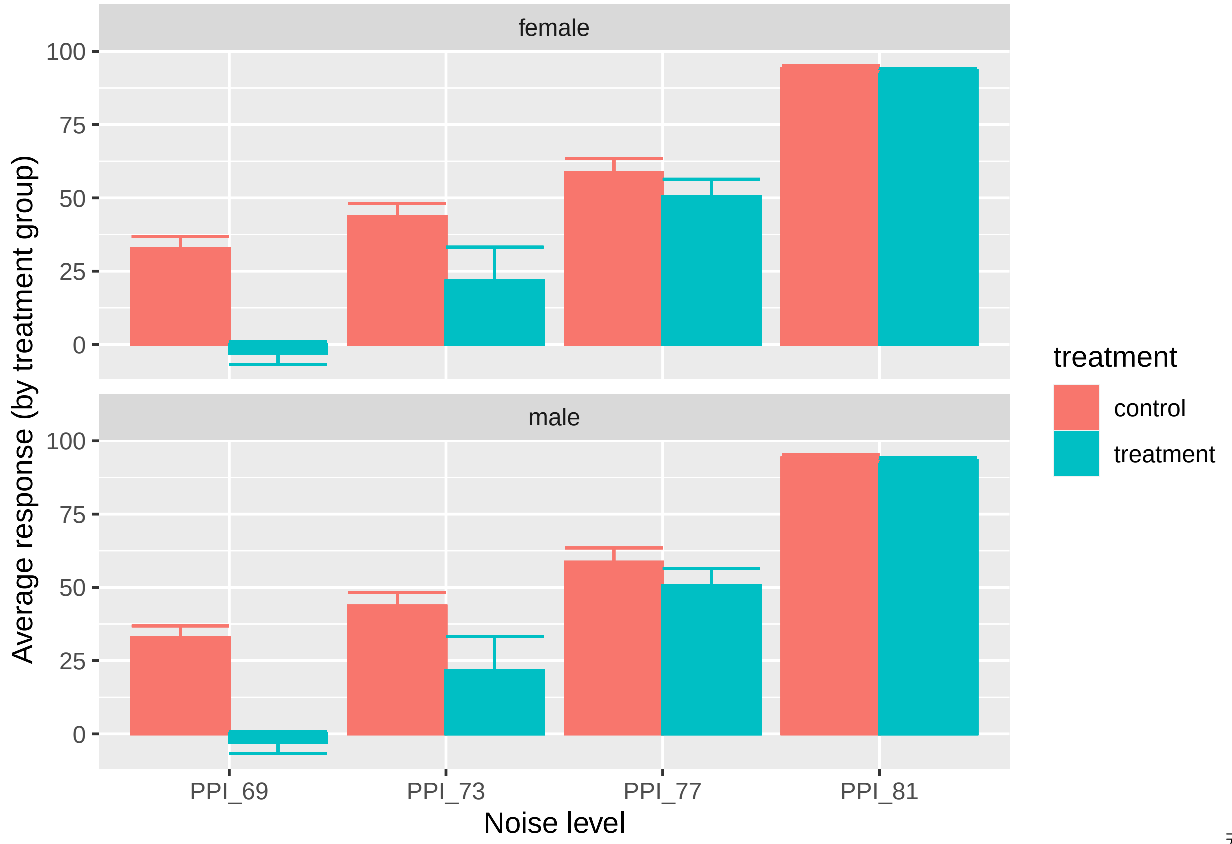

#

Statistical analysis We use mixed models, with random effect per mouse. The outcome is called "response" in the data, predictors are treatment vs control, exposure level, sex, and all possible interactions.

ANOVA table

Generating an ANOVA table for the full model:

```
mod_treat <- lmer(response ~  as.factor(sex)*as.factor(level)*as.factor(treatment) +
                                                                  (1 | subj), dat)
anova_table <- round(anova(mod_treat),4)
rownames(anova_table) <- sub("as.factor(level)", "level", rownames(anova_table),
                                                                 fixed = TRUE)
rownames(anova_table) <- sub("as.factor(sex)", "sex", rownames(anova_table),
                                                               fixed = TRUE)
rownames(anova_table) <- sub("as.factor(treatment)", "treatment", rownames(anova_table),
                                                                       fixed = TRUE)
anova_table
```

```
## Type III Analysis of Variance Table with Satterthwaite's method
##                        Sum Sq Mean Sq NumDF DenDF F value Pr(>F)
## sex                       324   323.9     1    23  0.8222 0.3739
## level                   89631 29877.1     3    69 75.8453 <2e-16 ***
## treatment                5303  5302.6     1    23 13.4609 0.0013 **
## sex:level                 546   181.9     3    69  0.4618 0.7099
```

```
## sex:treatment          324   323.9     1    23  0.8222 0.3739
## level:treatment       4809  1603.1     3    69  4.0697 0.0101 *
## sex:level:treatment    546   181.9     3    69  0.4618 0.7099
## ---
## Signif. codes:  0 '***' 0.001 '**' 0.01 '*' 0.05 '.' 0.1 ' ' 1
```

There is no evidence of sex effect.

Female-only analysis

ANOVA table

ANOVA table for the full model:

```
mod_treat <- lmer(response ~  as.factor(level)*as.factor(treatment) +  (1 | subj),
                                                            dat[sex == "female"])
anova_table <- round(anova(mod_treat),4)
rownames(anova_table) <- sub("as.factor(level)", "level", rownames(anova_table),
                                                                    fixed = TRUE)
rownames(anova_table) <- sub("as.factor(treatment)", "treatment", rownames(anova_table),
                                                                      fixed = TRUE)
anova_table
```

```
## Type III Analysis of Variance Table with Satterthwaite's method
##                 Sum Sq Mean Sq NumDF DenDF F value Pr(>F)
## level            54226 18075.4     3    39 48.2678 <2e-16 ***
## treatment         1620  1620.5     1    13  4.3273 0.0579 .
## level:treatment   2682   894.0     3    39  2.3872 0.0837 .
## ---
## Signif. codes:  0 '***' 0.001 '**' 0.01 '*' 0.05 '.' 0.1 ' ' 1
```

Female-specific effect estimates

```
effects <- summary(mod_treat)$coef
rownames(effects) <- sub("as.factor(level)", "", rownames(effects), fixed = TRUE)
rownames(effects) <- sub("as.factor(treatment)", "", rownames(effects), fixed = TRUE)
round(effects,4)
```

```
##                   Estimate Std. Error      df t value Pr(>|t|)
## (Intercept)        28.9154     7.3586 49.2844  3.9295   0.0003
## PPI_73             12.9265     9.6758 39.0000  1.3360   0.1893
## PPI_77             21.0174     9.6758 39.0000  2.1722   0.0360
## PPI_81             66.1730     9.6758 39.0000  6.8390   0.0000
## treatment         -31.8481    10.7718 49.2844 -2.9566   0.0048
## PPI_73:treatment   11.6700    14.1639 39.0000  0.8239   0.4150
## PPI_77:treatment   32.4084    14.1639 39.0000  2.2881   0.0276
## PPI_81:treatment   30.1635    14.1639 39.0000  2.1296   0.0396
```

Male-only analysis

ANOVA table

ANOVA table for the full model:

```r
mod_treat <- lmer(response ~  as.factor(level)*as.factor(treatment) +  (1 | subj),
                                                                   dat[sex == "male"])
anova_table <- round(anova(mod_treat),4)
rownames(anova_table) <- sub("as.factor(level)", "level", rownames(anova_table),
                                                                 fixed = TRUE)
rownames(anova_table) <- sub("as.factor(treatment)", "treatment", rownames(anova_table),
                                                                       fixed = TRUE)
anova_table
```

```
## Type III Analysis of Variance Table with Satterthwaite's method
##                  Sum Sq Mean Sq NumDF DenDF F value Pr(>F)
## level             37950 12649.9     3    30 30.1767 <2e-16 ***
## treatment          3934  3933.8     1    10  9.3841 0.0120 *
## level:treatment    2674   891.4     3    30  2.1265 0.1177
## ---
## Signif. codes:  0 '***' 0.001 '**' 0.01 '*' 0.05 '.' 0.1 ' ' 1
```

Male-specific effect estimates

```r
effects <- summary(mod_treat)$coef
rownames(effects) <- sub("as.factor(level)", "", rownames(effects), fixed = TRUE)
rownames(effects) <- sub("as.factor(treatment)", "", rownames(effects), fixed = TRUE)
round(effects,4)
```

```
##                    Estimate Std. Error     df t value Pr(>|t|)
## (Intercept)         38.9062     9.6435 38.869  4.0344   0.0002
## PPI_73               7.6330    12.9491 30.000  0.5895   0.5600
## PPI_77              33.6145    12.9491 30.000  2.5959   0.0145
## PPI_81              53.8035    12.9491 30.000  4.1550   0.0002
## treatment          -41.8388    12.6264 38.869 -3.3136   0.0020
## PPI_73:treatment    16.9635    16.9543 30.000  1.0005   0.3250
## PPI_77:treatment    19.8113    16.9543 30.000  1.1685   0.2518
## PPI_81:treatment    42.5330    16.9543 30.000  2.5087   0.0178
```

Statistical analysis amphetamine incudce activity experiment

Contents

Read the data **1**

Make sure we have the right numbers of mice per group: **1**

Visualize the data **1**

Statistical analysis **4**
Mixed model with mouse-specific intercept 4

ANOVA table **4**

Time intervals-specific effects **5**

Male-only analysis **5**

Female-only analysis **6**

Read the data

```
data_file_name <- "amphetamin_data.csv"
dat <- read.csv(data_file_name)
```

Make sure we have the right numbers of mice per group:

```
table(dat[which(dat$time == 1),c("sex", "status")])
```

```
##          status
## sex       control treatment
##   female        8         7
##   male          5         7
```

Visualize the data

First, we look at the distance moved in each 5 minute block by each of the mice.

```
dat <- data.table(dat) ## working with data.table is easier
dat[,sex_status := paste0(sex, "_", status)]

### figure of all individual mice movement in time:
p <- ggplot(data=dat, aes(x=time, y=move, group=subj, color = sex_status)) +
  geom_line()+
  geom_point() +
  xlab("Number of 5 minute block") +
```

```
  ylab("Distance moved in time block")
p + facet_wrap(~sex, ncol = 1)
```

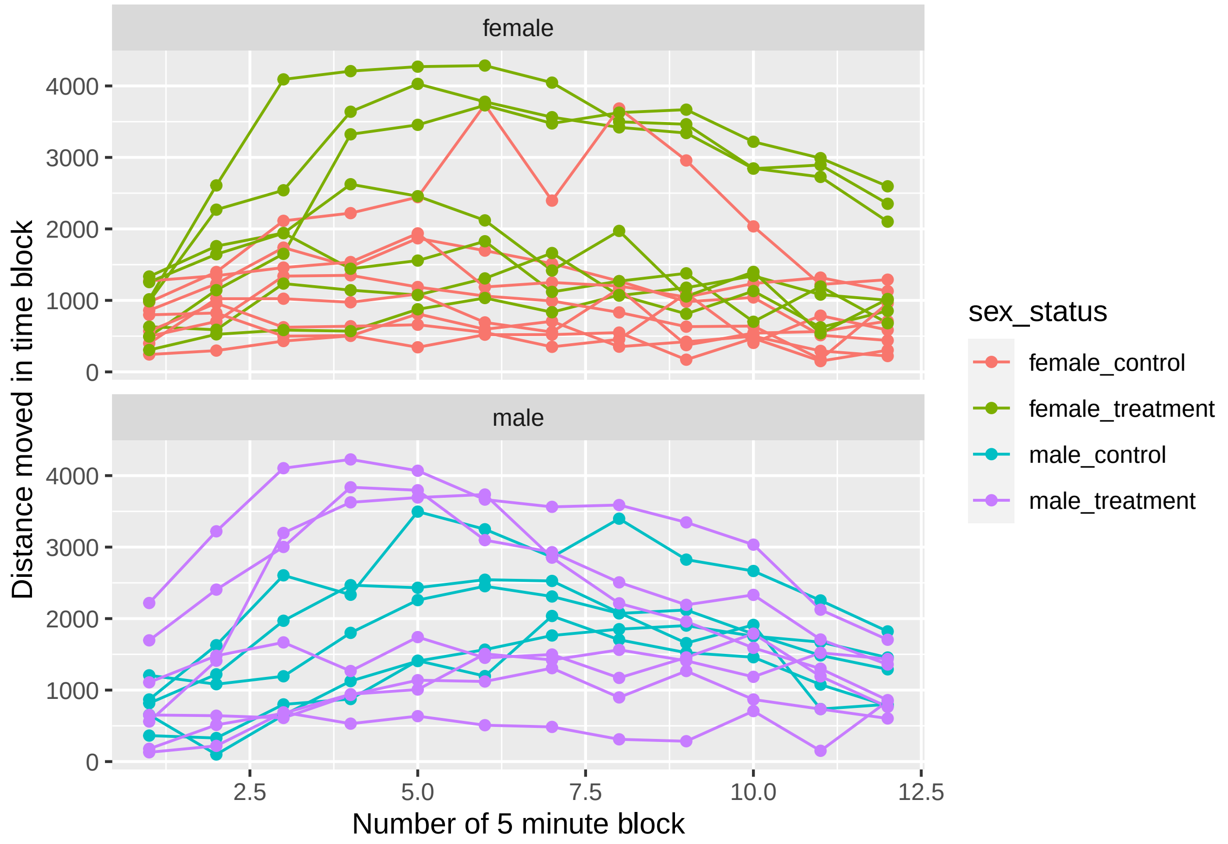

Second, we look at the distance moved in each 5 minute block, averaged across mice in the treatment and control groups. We add error bars around the mean movement in the groups. Length of the bars is 2 times the standard errors of the movement computed over the mice in the group, at the given time point.

```
### movement averaged across groups treatment only:
dat[,mean_move_time := mean(move), by = c("time", "status")]
dat[,low_bar := mean(move) - sd(move)/sqrt(length(move)), by = c("time", "status")]
dat[,high_bar := mean(move) + sd(move)/sqrt(length(move)), by = c("time", "status")]
p <- ggplot(data=dat, aes(x=time, y=mean_move_time, group=status, color = status)) +
  geom_line()+
  geom_point() +
  geom_errorbar(aes(x = time, ymin = low_bar, ymax = high_bar)) +
  xlab("Number of 5 minute block") +
  ylab("Average distance (in treatment group) moved in time block")
p
```

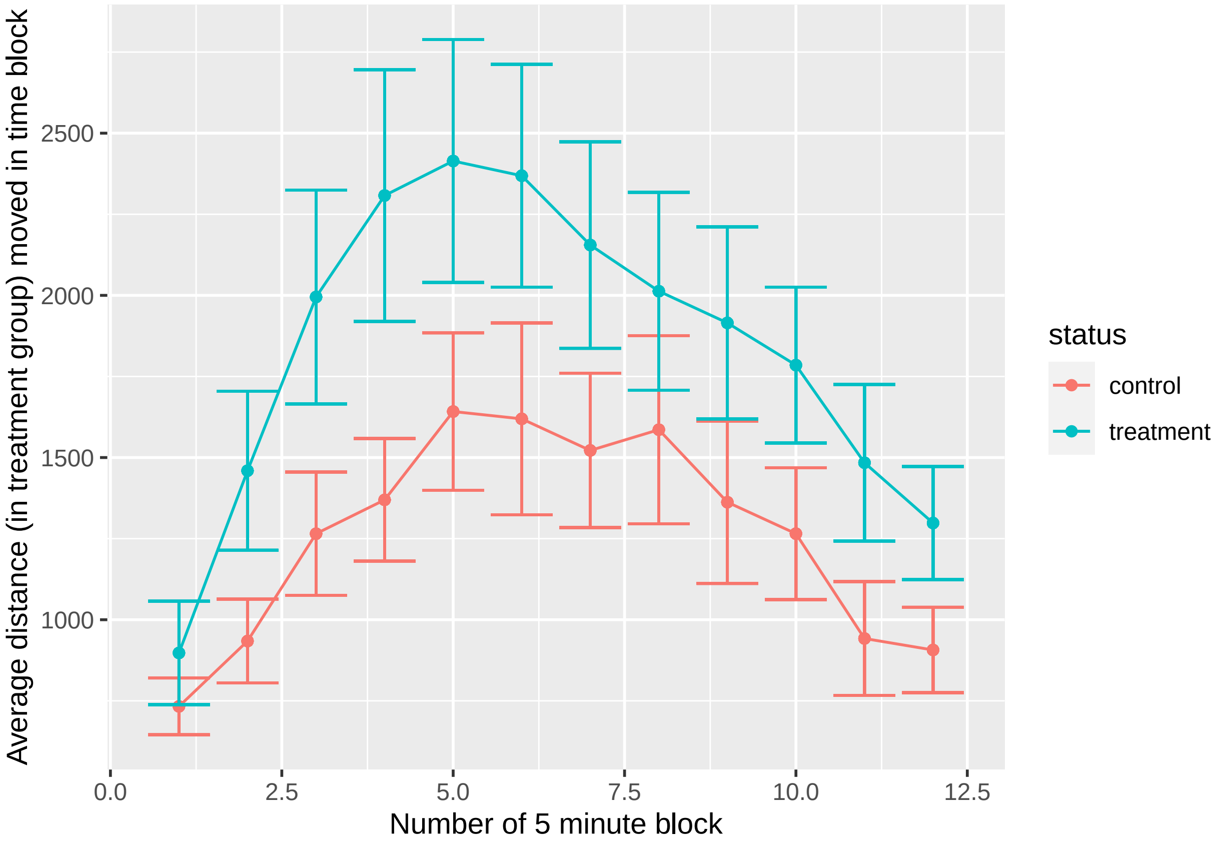

A similar figure, Astratified by sex:

```
dat[,mean_move_time := mean(move), by = c("time", "status", "sex")]
dat[,low_bar := mean(move) - sd(move)/sqrt(length(move)), by = c("time", "status", "sex")]
dat[,high_bar := mean(move) + sd(move)/sqrt(length(move)), by = c("time", "status", "sex")]
### movement averaged across groups defined by sex and treatment:
p <- ggplot(data=dat, aes(x=time, y=mean_move_time, group=sex_status, color = sex_status)) +
  geom_line()+
  geom_point() +
  geom_errorbar(aes(x = time, ymin = low_bar, ymax = high_bar)) +
  xlab("Number of 5 minute block") +
  ylab("Average distance (in sex/treatment group) moved in time block")
p + facet_wrap(~sex, ncol = 1)
```

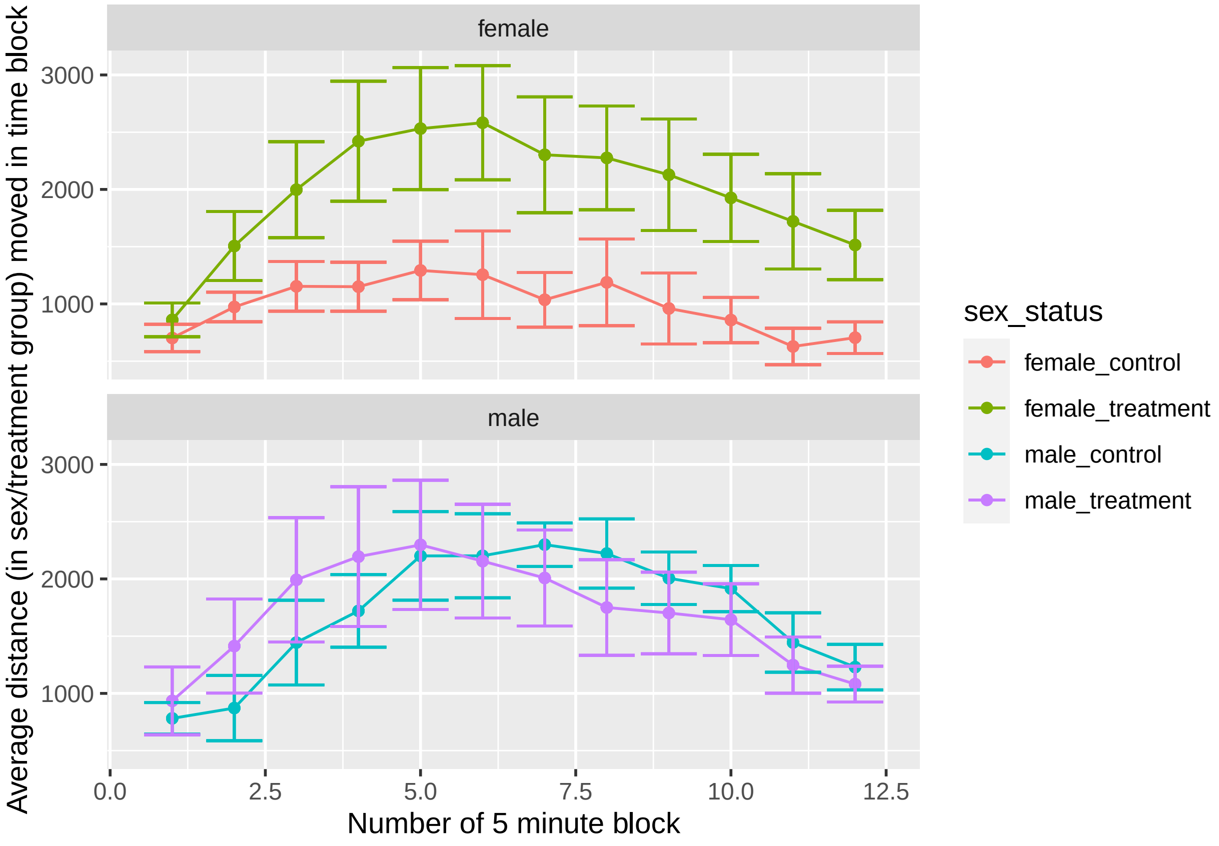

Statistical analysis

First, we explain the rationale for the model, then provide the code and results.

Mixed model with mouse-specific intercept

We have 12 repeated measures for each of the mice (average distance moved in 5 minutes). We will assume that each mice have an individual effect, which can be interpreted as a mouse-specific average distance moved in every 5 minutes block. This is modelled in a mixed model with mouse-specific intercept, under the standard assumption that these random effects come from a normal distribution. This modeling assumption is made in the code using the (1|subj) of the model statement seen later.

In the following code, we fit a baseline model, without treatment effect, and then a model with treatment effect. We then compare the two models using the anova command.

ANOVA table

```
dat[,treated := as.numeric(status == "treatment")]
dat[,male := as.numeric(sex == "male")]
mod_treat <- lmer(move ~  treated*male  +  (1 | subj), dat)
anova(mod_treat)
```

```
## Type III Analysis of Variance Table with Satterthwaite's method
##                Sum Sq Mean Sq NumDF DenDF F value  Pr(>F)
## treated       2008819 2008819     1    23  5.3813 0.02958 *
## male           836425  836425     1    23  2.2406 0.14802
```

```
## treated:male  868755  868755     1    23  2.3273 0.14076
## ---
## Signif. codes:  0 '***' 0.001 '**' 0.01 '*' 0.05 '.' 0.1 ' ' 1
```

and the effect estimates:

```
round(summary(mod_treat)$coeff, 3)
```

```
##              Estimate Std. Error df  t value Pr(>|t|)
## (Intercept)   992.072    291.099 23   3.408    0.002
## treated       988.511    426.126 23   2.320    0.030
## male          702.610    469.384 23   1.497    0.148
## treated:male -981.583    643.436 23  -1.526    0.141
```

Time intervals-specific effects

There is some interest in estimating the effect in each time intervals in the first hour. Therefore, we will create a facor with levels for each of the 12 5-minutes time intervals and repeat the analysis.

```
dat[,time_interval := paste0("interval_", time) ]
dat$time_interval <- factor(dat$time_interval, levels = c(paste0("interval_", 1:12)))
mod_treat <- lmer(move ~  male*treated*time_interval + (1 | subj), dat)
anova(mod_treat)
```

```
## Type III Analysis of Variance Table with Satterthwaite's method
##                              Sum Sq Mean Sq NumDF DenDF F value
## male                         486378  486378     1    23  2.2406
## treated                     1168121 1168121     1    23  5.3813
## time_interval               4551221  413747    11   253  1.9060
## male:treated                 505178  505178     1    23  2.3272
## male:time_interval          6261767  569252    11   253  2.6224
## treated:time_interval       5013758  455796    11   253  2.0998
## male:treated:time_interval 6132500  557500    11   253  2.5683
##                              Pr(>F)
## male                       0.148023
## treated                    0.029584 *
## time_interval              0.038969 *
## male:treated               0.140761
## male:time_interval         0.003478 **
## treated:time_interval      0.020829 *
## male:treated:time_interval 0.004208 **
## ---
## Signif. codes:  0 '***' 0.001 '**' 0.01 '*' 0.05 '.' 0.1 ' ' 1
```

Male-only analysis

```
dat[,time_interval := paste0("interval_", time) ]
dat$time_interval <- factor(dat$time_interval, levels = c(paste0("interval_", 1:12)))
mod_treat <- lmer(move ~  treated*time_interval + (1 | subj), dat[sex == "male"])
anova(mod_treat)
```

```
## Type III Analysis of Variance Table with Satterthwaite's method
##                         Sum Sq Mean Sq NumDF DenDF F value    Pr(>F)
## treated                     41      41     1    10  0.0002   0.98913
## time_interval         15776546 1434231    11   110  6.7849 1.318e-08
```

```
## treated:time_interval  4043723  367611    11   110  1.7391   0.07398
##
## treated
## time_interval         ***
## treated:time_interval .
## ---
## Signif. codes:  0 '***' 0.001 '**' 0.01 '*' 0.05 '.' 0.1 ' ' 1
```

Female-only analysis

```r
dat[,time_interval := paste0("interval_", time) ]
dat$time_interval <- factor(dat$time_interval, levels = c(paste0("interval_", 1:12)))
mod_treat <- lmer(move ~  treated*time_interval + (1 | subj), dat[sex == "female"])
anova(mod_treat)
```

```
## Type III Analysis of Variance Table with Satterthwaite's method
##                        Sum Sq Mean Sq NumDF DenDF F value  Pr(>F)
## treated               1247533 1247533     1    13  5.6336 0.03371 *
## time_interval         4551221  413747    11   143  1.8684 0.04821 *
## treated:time_interval 5013758  455796    11   143  2.0583 0.02709 *
## ---
## Signif. codes:  0 '***' 0.001 '**' 0.01 '*' 0.05 '.' 0.1 ' ' 1
```

Statistical analysis of habituation period

Contents

Read the data 1

Make sure we have the right numbers of mice per group: 1

Visualize the data 1

Mixed model with mouse-specific intercept 4

Check if there is a significant treatment effect 4

ANOVA table 5

Read the data

```
data_file_name <- "habituation_30_min.csv"
dat <- read.csv(data_file_name)
```

Make sure we have the right numbers of mice per group:

```
table(dat[which(dat$time == 1),c("sex", "status")])
```

```
##         status
## sex       control treatment
##   female       8         7
##   male         5         7
```

Visualize the data

First, we look at the distance moved in each 5 minute block by each of the mice.

```
dat <- data.table(dat) ## working with data.table is easier
dat[,sex_status := paste0(sex, "_", status)]

### figure of all individual mice movement in time:
p <- ggplot(data=dat, aes(x=time, y=move, group=subj, color = sex_status)) +
  geom_line()+
  geom_point() +
  xlab("Number of 5 minute block") +
  ylab("Distance moved in time block")
p + facet_wrap(~sex, ncol = 1)
```

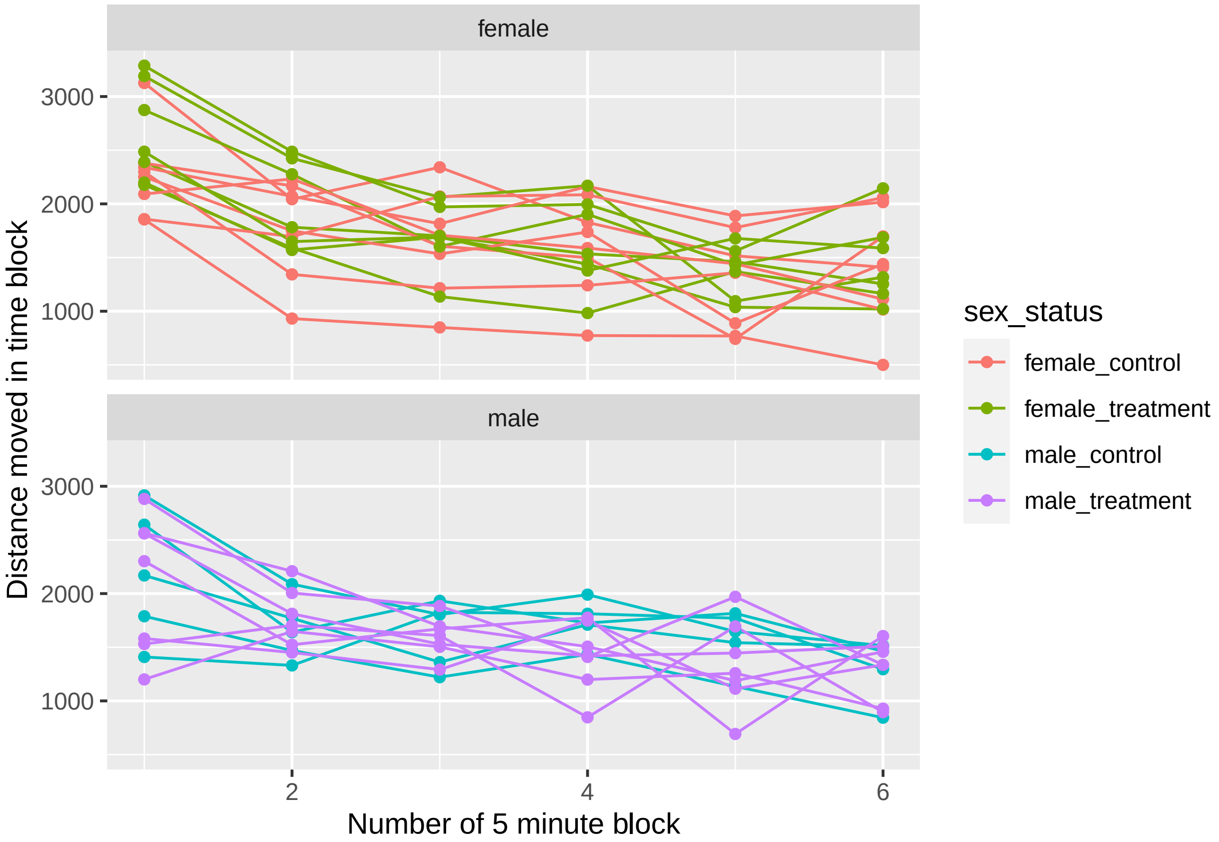

Second, we look at the distance moved in each 5 minute block, averaged across mice in each of the treatment group. We now add bars around the mean movement in the groups. The length of the bars is 2 standard errors of the movement computed over the mice in the group.

```
### movement averaged across groups treatment only:
dat[,mean_move_time := mean(move), by = c("time", "status")]
dat[,low_bar := mean(move) - sd(move)/sqrt(length(move)), by = c("time", "status")]
dat[,high_bar := mean(move) + sd(move)/sqrt(length(move)), by = c("time", "status")]
p <- ggplot(data=dat, aes(x=time, y=mean_move_time, group=status, color = status)) +
  geom_line()+
  geom_point() +
  geom_errorbar(aes(x = time, ymin = low_bar, ymax = high_bar)) +
  xlab("Number of 5 minute block") +
  ylab("Average distance (in treatment group) moved in time block")
p
```

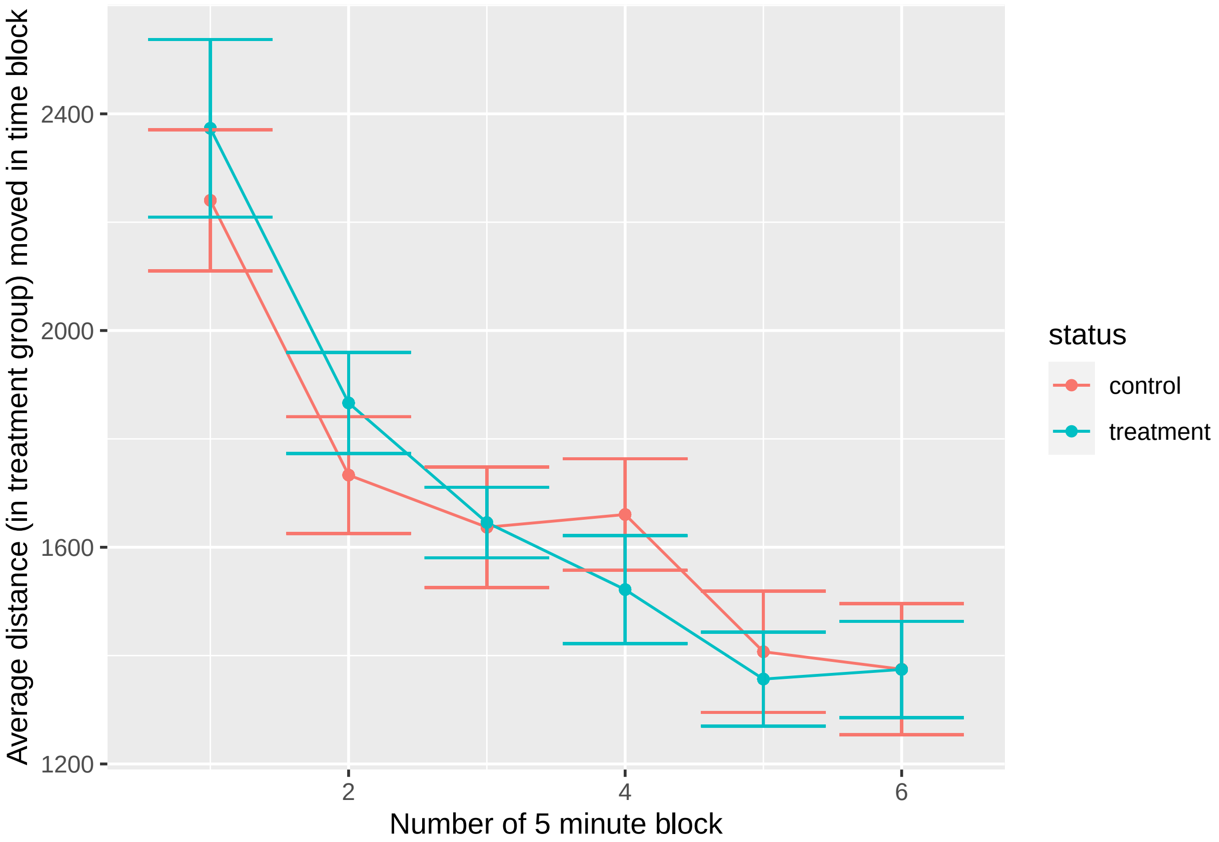

And stratified by sex:

```
dat[,mean_move_time := mean(move), by = c("time", "status", "sex")]
dat[,low_bar := mean(move) - sd(move)/sqrt(length(move)), by = c("time", "status", "sex")]
dat[,high_bar := mean(move) + sd(move)/sqrt(length(move)), by = c("time", "status", "sex")]
### movement averaged across groups defined by sex and treatment:
p <- ggplot(data=dat, aes(x=time, y=mean_move_time, group=sex_status, color = sex_status)) +
  geom_line()+
  geom_point() +
  geom_errorbar(aes(x = time, ymin = low_bar, ymax = high_bar)) +
  xlab("Number of 5 minute block") +
  ylab("Average distance (in sex/treatment group) moved in time block")
p + facet_wrap(~sex, ncol = 1)
```

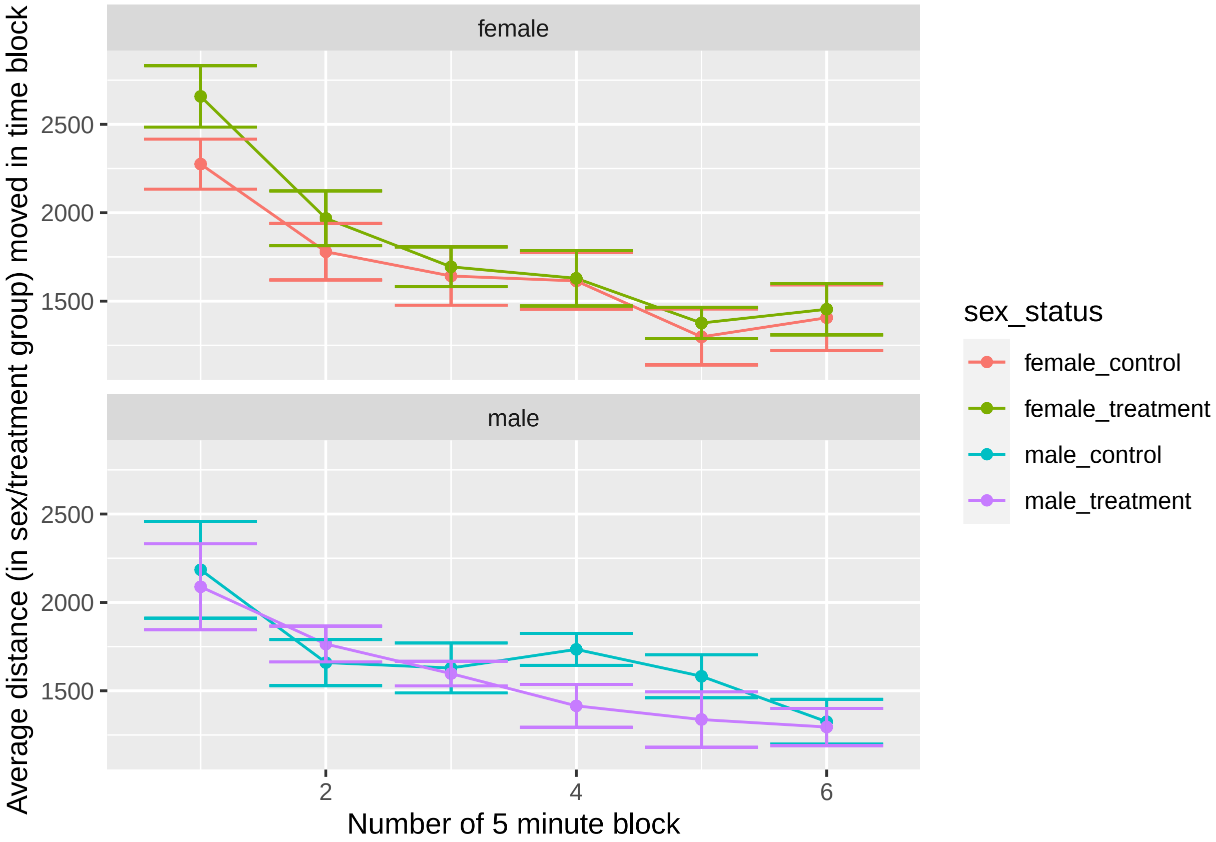

Mixed model with mouse-specific intercept

We have 6 repeated measures for each of the mice (average distance moved in 5 minutes). We assume that each mice have an individual effect, which can be interpreted as a mouse-specific average distance moved in every 5 minutes block. This is modeled in a mixed model with mouse-specific intercept, under the standard assumption that these random effects come from a normal distribution. This modeling assumption is made in the code using the (1|subj) of the model statement seen later.

In the following code, we fit a baseline model, without treatment effect, and then a model with treatment effect in the first hour. We then compare the two models using the anova command.

Check if there is a significant treatment effect

We do this by comparing a model with treatment effect (and treatment-sex interaction), to a model without

```
dat[,treated := as.numeric(status == "treatment")]
dat[,male := as.numeric(sex == "male")]
dat[,time_interval := paste0("interval_", time) ]
dat$time_interval <- factor(dat$time_interval, levels = c(paste0("interval_", 1:6)))
mod_treat <- lmer(move ~  treated*male*time_interval  +  (1 | subj), dat)
mod_notreat <- lmer(move ~  male*time_interval  +  (1 | subj), dat)
anova(mod_treat, mod_notreat)
```

```
## refitting model(s) with ML (instead of REML)
```

```
## Data: dat
## Models:
```

```
## mod_notreat: move ~ male * time_interval + (1 | subj)
## mod_treat: move ~ treated * male * time_interval + (1 | subj)
##             npar    AIC    BIC  logLik deviance  Chisq Df Pr(>Chisq)
## mod_notreat   14 2359.9 2403.2 -1166.0   2331.9
## mod_treat     26 2373.2 2453.5 -1160.6   2321.2 10.742 12     0.5511
```

There is no evidence for treatment effect (the model that includes a treatment effect does not have a better fit compared to a model that does not model a treatment effect).

ANOVA table

```
anova(mod_treat)
```

```
## Type III Analysis of Variance Table with Satterthwaite's method
##                              Sum Sq Mean Sq NumDF DenDF F value
## treated                       56253   56253     1    23  0.6690
## male                            814     814     1    23  0.0097
## time_interval               4723327  944665     5   115 11.2347
## treated:male                  80632   80632     1    23  0.9589
## treated:time_interval        359355   71871     5   115  0.8547
## male:time_interval           378316   75663     5   115  0.8998
## treated:male:time_interval   241536   48307     5   115  0.5745
##                                Pr(>F)
## treated                        0.4218
## male                           0.9225
## time_interval               7.836e-09 ***
## treated:male                   0.3376
## treated:time_interval          0.5139
## male:time_interval             0.4838
## treated:male:time_interval     0.7194
## ---
## Signif. codes:  0 '***' 0.001 '**' 0.01 '*' 0.05 '.' 0.1 ' ' 1
```

Statistical analysis of saline induced activity

Contents

Read the data 1

Make sure we have the right numbers of mice per group: 1

Visiualize the data 1

Mixed model with mouse-specific intercept 4

ANOVA table 4

Time intervals-specific effects 5

Read the data

```
data_file_name <- "saline_30_min.csv"
dat <- read.csv(data_file_name)
```

Make sure we have the right numbers of mice per group:

```
table(dat[which(dat$time == 1),c("sex", "status")])
```

```
##         status
## sex       control treatment
##   female        8         7
##   male          5         7
```

Visiualize the data

First, we look at the distance moved in each 5 minute block by each of the mice.

```
dat <- data.table(dat) ## working with data.table is easier
dat[,sex_status := paste0(sex, "_", status)]

### figure of all individual mice movement in time:
p <- ggplot(data=dat, aes(x=time, y=move, group=subj, color = sex_status)) +
  geom_line()+
  geom_point() +
  xlab("Number of 5 minute block") +
  ylab("Distance moved in time block")
p + facet_wrap(~sex, ncol = 1)
```

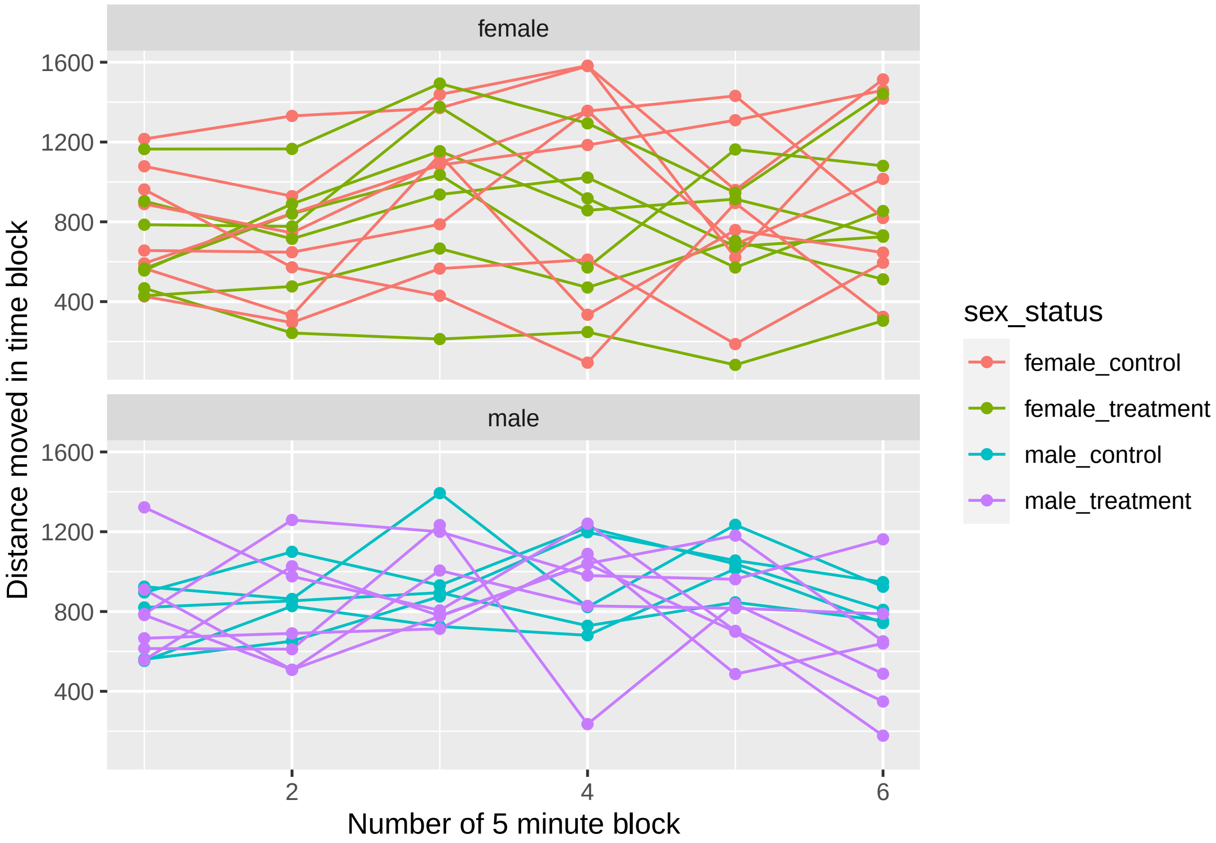

We now visualize the data by averaging across the treatment groups. We look at the distance moved in each 5 minute block, averaged across mice in the groups. We add error bars centered at each mean. The length of a bar is 2 times the standard errors of the mean, computed over the mice in the group at that time point.

```
dat[,mean_move_time := mean(move), by = c("time", "status")]
dat[,low_bar := mean(move) - sd(move)/sqrt(length(move)), by = c("time", "status")]
dat[,high_bar := mean(move) + sd(move)/sqrt(length(move)), by = c("time", "status")]
p <- ggplot(data=dat, aes(x=time, y=mean_move_time, group=status, color = status)) +
  geom_line()+
  geom_point() +
  geom_errorbar(aes(x = time, ymin = low_bar, ymax = high_bar)) +
  xlab("Number of 5 minute block") +
  ylab("Average distance (in treatment group) moved in time block")
p
```

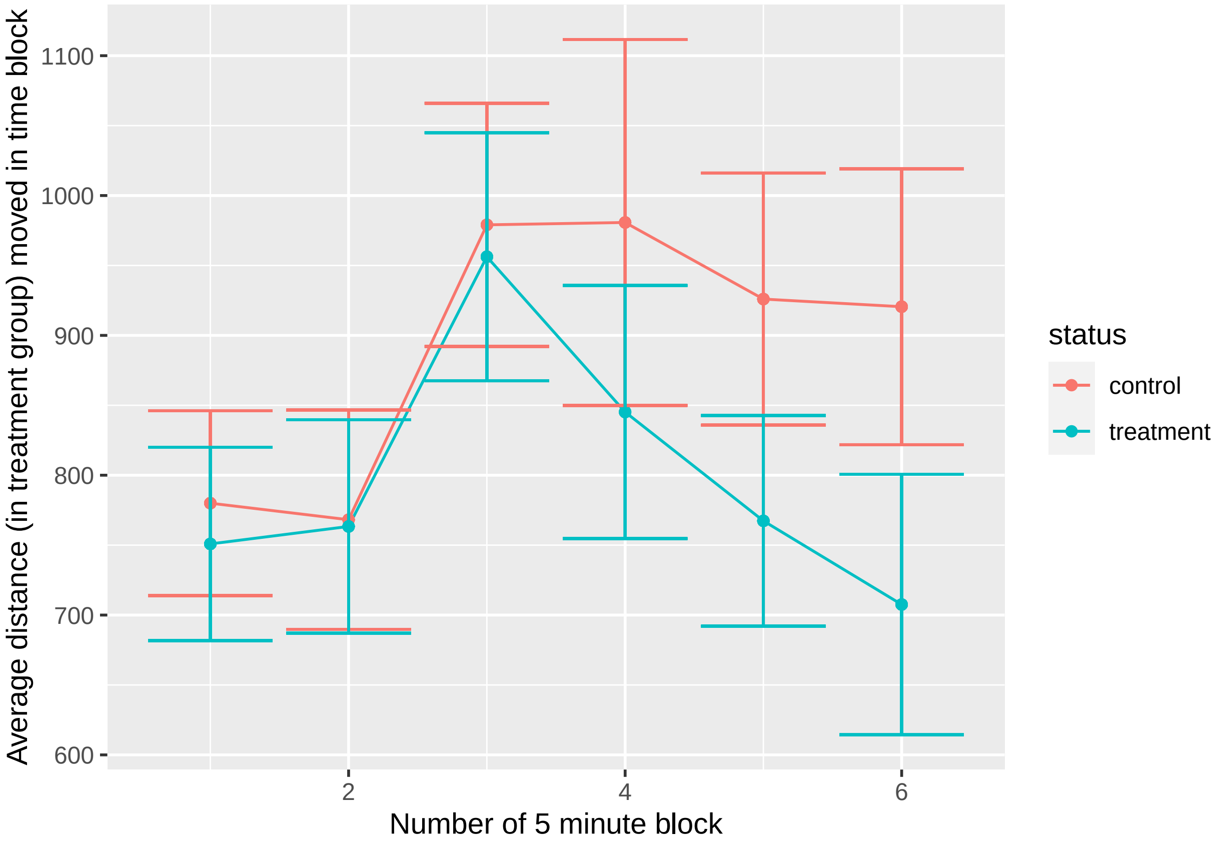

And sex stratified:

```
dat[,mean_move_time := mean(move), by = c("time", "status", "sex")]
dat[,low_bar := mean(move) - sd(move)/sqrt(length(move)), by = c("time", "status", "sex")]
dat[,high_bar := mean(move) + sd(move)/sqrt(length(move)), by = c("time", "status", "sex")]
### movement averaged across groups defined by sex and treatment:
p <- ggplot(data=dat, aes(x=time, y=mean_move_time, group=sex_status, color = sex_status)) +
  geom_line()+
  geom_point() +
  geom_errorbar(aes(x = time, ymin = low_bar, ymax = high_bar)) +
  xlab("Number of 5 minute block") +
  ylab("Average distance (in sex/treatment group) moved in time block")
p + facet_wrap(~sex, ncol = 1)
```

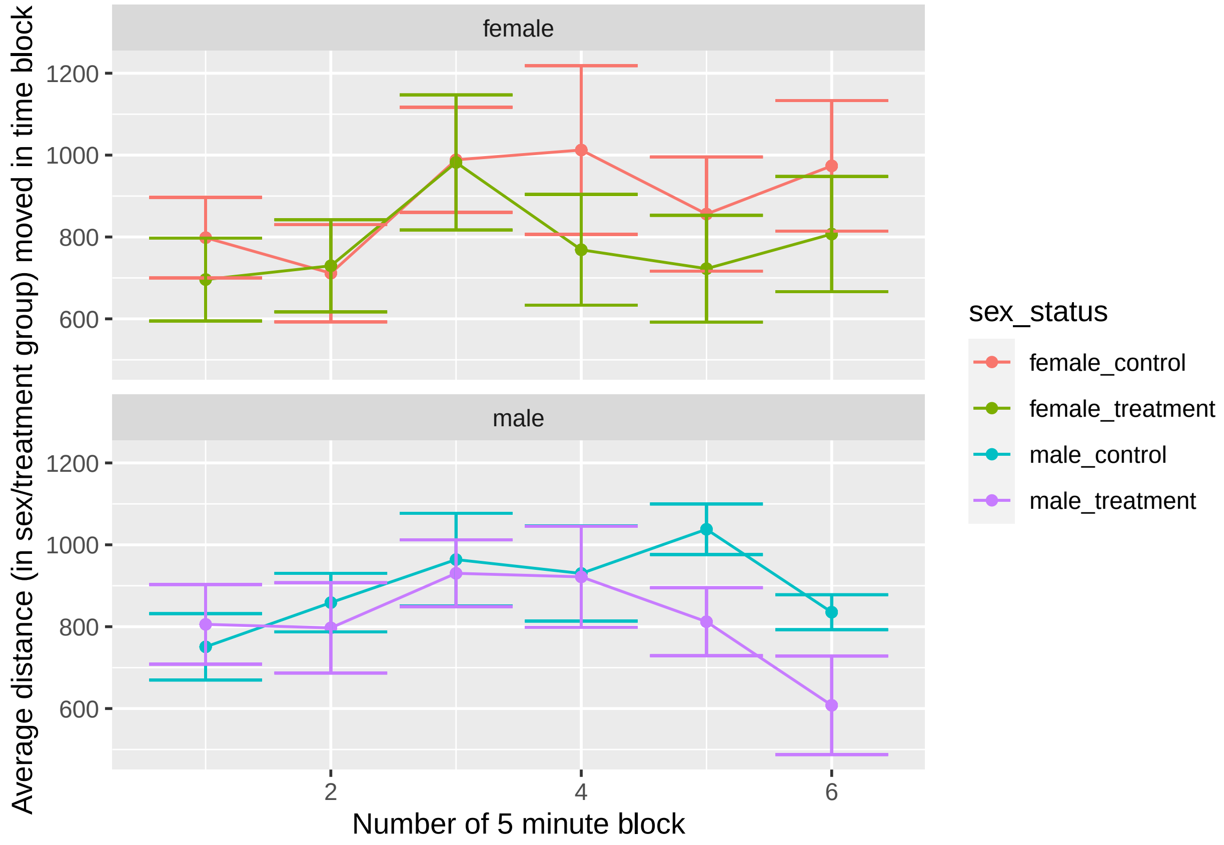

Mixed model with mouse-specific intercept

We have 6 repeated measures for each of the mice (each representing the average distance moved in 5 minutes). We assume that each mice have an individual effect, which can be interpreted as a mouse-specific average distance moved in every 5 minutes block. This is modeled in a mixed model with mouse-specific intercept, under the standard assumption that these random effects come from a normal distribution. This modeling assumption is made in the code using the (1|subj) of the model statement seen later.

In the following code, we fit a baseline model, without treatment effect, and then a model with treatment effect in the first hour. We then compare the two models using the anova command.

ANOVA table

```
dat[,treated := as.numeric(status == "treatment")]
dat[,male := as.numeric(sex == "male")]
mod_treat <- lmer(move ~  treated*male  +  (1 | subj), dat)
anova(mod_treat)
```

```
## Type III Analysis of Variance Table with Satterthwaite's method
##              Sum Sq Mean Sq NumDF DenDF F value Pr(>F)
## treated       38647   38647     1    23  0.6507 0.4281
## male            106     106     1    23  0.0018 0.9667
## treated:male    739     739     1    23  0.0124 0.9122
```

and the effect estimates:

```r
summary(mod_treat)$coeff
```

```
##                 Estimate Std. Error df     t value     Pr(>|t|)
## (Intercept)   890.065850   89.53582 23  9.94089080 8.548413e-10
## treated      -105.725369  131.06703 23 -0.80665116 4.281303e-01
## male            6.085117  144.37218 23  0.04214882 9.667438e-01
## treated:male   22.072045  197.90687 23  0.11152743 9.121657e-01
```

This suggest no treatment effect. There is potentially effect that we will see if we account for different movement by time, motivating the following model.

Time intervals-specific effects

Here, we account for potential differences in movement overtime, and potential treatmnet effect that varies by time as well. We create a factor variables with levels for each of the 12 5-minutes time intervals, and repeat the analysis above.

```r
dat[,time_interval := paste0("interval_", time) ]
dat$time_interval <- factor(dat$time_interval, levels = c(paste0("interval_", 1:12)))
mod_treat <- lmer(move ~  male*treated*time_interval + (1 | subj), dat)
anova(mod_treat)
```

```
## Type III Analysis of Variance Table with Satterthwaite's method
##                            Sum Sq Mean Sq NumDF DenDF F value  Pr(>F)
## male                           98      98     1    23  0.0018 0.96674
## treated                     35736   35736     1    23  0.6507 0.42813
## time_interval              584941  116988     5   115  2.1301 0.06673
## male:treated                  683     683     1    23  0.0124 0.91217
## male:time_interval         256533   51307     5   115  0.9342 0.46166
## treated:time_interval      181921   36384     5   115  0.6625 0.65263
## male:treated:time_interval 158120   31624     5   115  0.5758 0.71841
##
## male
## treated
## time_interval              .
## male:treated
## male:time_interval
## treated:time_interval
## male:treated:time_interval
## ---
## Signif. codes:  0 '***' 0.001 '**' 0.01 '*' 0.05 '.' 0.1 ' ' 1
```

There is no evidence of differences by treatment group.
