# Supplementary material for: Increased RNA editing in maternal immune activation model of neurodevelopmental disease
Source: Nat Commun. 2020 Oct 16;11:5236. doi: 10.1038/s41467-020-19048-6 (PMC7567798; doi:10.1038/s41467-020-19048-6)
Supplement: Supplementary file 2 — Description of Additional Supplementary Files [file 41467_2020_19048_MOESM2_ESM.pdf]

Description of Additional Supplementary Files

Title: Supplementary Data 1.

Description: Differential gene expression analysis (DESeq). Wald test followed by the Benjamini–Hochberg false discovery rate (FDR) multiple-testing.

Title: Supplementary Data 2.

Description: Gene set enrichment analysis (GSEA): up and down regulated gene sets.

Title: Supplementary Data 3.

Description: A full list of the gene sets included in Fig. 2d.

Title: Supplementary Data 4.

Description: Hyper editing A-to-G sites and refseq genes table.

Title: Supplementary Data 5.

Description: RNA editing levels in conserved coding sites in datasets from older PolyI:C mice

Title: Supplementary Data 6.

Description: RNA editing levels in conserved coding sites in datasets from ASD postmortem human brain.

Title: Supplementary Data 7.

Description: RNA editing levels in conserved coding sites in datasets from schizophrenic postmortem human brain.
