# Supplementary material for: Increased RNA editing in maternal immune activation model of neurodevelopmental disease
Source: Nat Commun. 2020 Oct 16;11:5236. doi: 10.1038/s41467-020-19048-6 (PMC7567798; doi:10.1038/s41467-020-19048-6)
Supplement: Supplementary file 10 — Reporting Summary [file 41467_2020_19048_MOESM10_ESM.pdf]

nature research

Corresponding author(s): Erez Y. Levanon and Daniel Offen

Last updated by author(s): Sep 3, 2020

# Reporting Summary

Nature Research wishes to improve the reproducibility of the work that we publish. This form provides structure for consistency and transparency in reporting. For further information on Nature Research policies, see our Editorial Policies and the Editorial Policy Checklist.

## Statistics

For all statistical analyses, confirm that the following items are present in the figure legend, table legend, main text, or Methods section.

| n/a | Confirmed | |
|---|---|---|
| ☐ | ☒ | The exact sample size (*n*) for each experimental group/condition, given as a discrete number and unit of measurement |
| ☐ | ☒ | A statement on whether measurements were taken from distinct samples or whether the same sample was measured repeatedly |
| ☐ | ☒ | The statistical test(s) used AND whether they are one- or two-sided<br>*Only common tests should be described solely by name; describe more complex techniques in the Methods section.* |
| ☐ | ☒ | A description of all covariates tested |
| ☐ | ☒ | A description of any assumptions or corrections, such as tests of normality and adjustment for multiple comparisons |
| ☐ | ☒ | A full description of the statistical parameters including central tendency (e.g. means) or other basic estimates (e.g. regression coefficient) AND variation (e.g. standard deviation) or associated estimates of uncertainty (e.g. confidence intervals) |
| ☐ | ☒ | For null hypothesis testing, the test statistic (e.g. *F*, *t*, *r*) with confidence intervals, effect sizes, degrees of freedom and *P* value noted<br>*Give P values as exact values whenever suitable.* |
| ☒ | ☐ | For Bayesian analysis, information on the choice of priors and Markov chain Monte Carlo settings |
| ☒ | ☐ | For hierarchical and complex designs, identification of the appropriate level for tests and full reporting of outcomes |
| ☒ | ☐ | Estimates of effect sizes (e.g. Cohen's *d*, Pearson's *r*), indicating how they were calculated |

*Our web collection on statistics for biologists contains articles on many of the points above.*

## Software and code

Policy information about availability of computer code

| | |
|---|---|
| Data collection | Public SRA data was downloaded using the SRA toolkit version 2.8.2-1 |
| Data analysis | FastQC version 0.10.1 was used for evaluation of transcriptomic data quality.<br>Duplicate reads were removed using PrinseqLite version 0.20.4.<br>STAR version 2.5.2b was used to align fastq data.<br>Transcript quantification was determined using Salmon version 0.11.2 following tximport R library version 1.12.3.<br>Differential gene expression was evaluated on Salmon transcript counts using DESeq version 1.24.0.<br>Editing levels were determined using REDITools version 1.0.4.<br>Alu Editing Index version 1.0 and Hyper-editing were computed as described in PMID 31636457 and 25158696, respectively.<br>Gene set enrichment analysis was performed with the GSEA algorithm version 4.0.2.<br>Clustering of gene sets was performed in Cytoscape version 3.4.<br>Statistical analyses as described in the text were computed using R version 3.5.1. |

For manuscripts utilizing custom algorithms or software that are central to the research but not yet described in published literature, software must be made available to editors and reviewers. We strongly encourage code deposition in a community repository (e.g. GitHub). See the Nature Research guidelines for submitting code & software for further information.

## Data

Policy information about availability of data

All manuscripts must include a data availability statement. This statement should provide the following information, where applicable:

- Accession codes, unique identifiers, or web links for publicly available datasets
- A list of figures that have associated raw data
- A description of any restrictions on data availability

RNA-seq data of PolyI:C-treated mice and controls were generated in this study and have been deposited in the SRA (BioProject ID: PRJNA602886).
Additional RNA-seq datasets of PolyI:C-treated mice were downloaded from the SRA database (ERP014069, SRP221742).
RNA-seq datasets of schizophrenic human brain were obtained from the SRA database (SRP102186, SRP073813).
RNA-seq dataset of ASD human brain was obtained from PsychENCODE portal (syn4587615).
RNA-seq datasets of healthy human brain were obtained from the GTEx project via dbGaP (phs000424.v8.p2).
RNA-seq dataset of fetal mouse brain development was downloaded from the ArrayExpress Archive (E-MTAB-6798).

# Field-specific reporting

Please select the one below that is the best fit for your research. If you are not sure, read the appropriate sections before making your selection.

[x] Life sciences  [ ] Behavioural & social sciences  [ ] Ecological, evolutionary & environmental sciences

For a reference copy of the document with all sections, see nature.com/documents/nr-reporting-summary-flat.pdf

# Life sciences study design

All studies must disclose on these points even when the disclosure is negative.

**Sample size**

Previous studies (Roth et al.) have demonstrated that fewer than n=5 per group is sufficient for achieving significance in RNA-editing analysis. Based on average litter size of 5, we aimed for two pregnant dams in each group.
For behavioral tests, to assess the required number of mice for achieving 80% power, we used the function pwr.anova.test from the R "pwr" package. Based on two groups (treatment and control), significance level of 0.05, and effect size of 0.8 standard deviation of the outcome in the control mice, the required number of mice in each group was 7.2. We looked at a manuscript by Yee et al. (below), which reported a similar experiment. They observed effects sizes of more than 3 times the standard deviation of the outcome in the control mice (Figure 1 there), and thus concluded that at least 8 mice in each group is sufficient. Therefore we aimed for two pregnant dams in each group.

Roth SH, Levanon EY, Eisenberg E. Genome-wide quantification of ADAR adenosine-to-inosine RNA editing activity. Nat Methods. 2019;16 (11):1131-1138. doi:10.1038/s41592-019-0610-9

Yee, Benjamin K., DL Tilly Chang, and Joram Feldon. "The effects of dizocilpine and phencyclidine on prepulse inhibition of the acoustic startle reflex and on prepulse-elicited reactivity in C57BL6 mice." Neuropsychopharmacology 29.10 (2004): 1865-1877.

**Data exclusions**

No data was excluded.

**Replication**

All experiments were performed according to established protocols, previously published by different groups. No data or animals were excluded from the analysis. All details regarding the different experimental procedures are provided in the manuscript. The code for the statistical analysis of the behavioral experiments is provided as well.

**Randomization**

Animals were allocated at random for each experimental group.

**Blinding**

Behavioral experiments took place several months after birth, at which time they were separated by group. Behavioral tests and sata analysis were performed by different investigators. The data acquisition was completely automatic and gene expression levels as well as editing levels of each mouse were calculated independently and blindly.

# Reporting for specific materials, systems and methods

We require information from authors about some types of materials, experimental systems and methods used in many studies. Here, indicate whether each material, system or method listed is relevant to your study. If you are not sure if a list item applies to your research, read the appropriate section before selecting a response.

## Materials & experimental systems

| n/a | Involved in the study | |
| --- | --- | --- |
| ☒ | ☐ | Antibodies |
| ☒ | ☐ | Eukaryotic cell lines |
| ☒ | ☐ | Palaeontology and archaeology |
| ☐ | ☒ | Animals and other organisms |
| ☒ | ☐ | Human research participants |
| ☒ | ☐ | Clinical data |
| ☒ | ☐ | Dual use research of concern |

## Methods

| n/a | Involved in the study | |
| --- | --- | --- |
| ☒ | ☐ | ChIP-seq |
| ☒ | ☐ | Flow cytometry |
| ☒ | ☐ | MRI-based neuroimaging |

# Animals and other organisms

Policy information about studies involving animals; ARRIVE guidelines recommended for reporting animal research

| | |
| --- | --- |
| Laboratory animals | The study included C57BL/6 mice if both sexes. 8-12 week old pregnant dams were used, as well as mice embryos and mice which were reared from birth to 16 weeks of age. |
| Wild animals | The study did not involve wild animals. |
| Field-collected samples | The study did not involve samples collected from the field. |
| Ethics oversight | All experimental manipulations were performed in accordance with guidelines and regulations of the Tel Aviv University Committee of Animal Use for Research and Education. |

Note that full information on the approval of the study protocol must also be provided in the manuscript.
